# A two-year study of *Salmonella* in four natural watersheds highlights the need for increased environmental *Salmonella* surveillance to close the One Health loop

**DOI:** 10.1128/aem.01770-25

**Published:** 2025-12-04

**Authors:** Jared C. Smith, Amy T. Siceloff, Sherwin M. Shirazi, Rebecca L. Bell, Nikki W. Shariat

**Affiliations:** 1 Department of Population Health, College of Veterinary Medicine, University of Georgia 308501, Athens, Georgia, USA; 2 Department of Microbiology, College of Arts and Sciences, University of Georgia 189270, Athens, Georgia, USA; 3 Department of Statistics, College of Arts and Sciences, University of Georgia 138572, Athens, Georgia, USA; 4 Divison of Food and Environmental Safety, Office of Applied Microbiology and Technology, Office of Laboratory Operations and Applied Science, Human Foods Program, U.S. Food and Drug Administration 116043 , College Park, Maryland, USA; Centers for Disease Control and Prevention, Atlanta, Georgia, USA

**Keywords:** *Salmonella enterica*, surface water, One Health, environmental microbiology

## Abstract

*Salmonella enterica* is a leading bacterial cause of foodborne illness, often transmitted through contaminated food and water. Improved food handling has led to considerable reductions of *Salmonella* contamination in meat and poultry products; this does not wholly contribute to decreased salmonellosis incidence, highlighting the need to define alternative reservoirs and transmission pathways. In this study, we collected samples from four distinct watersheds over 24 months to characterize *Salmonella* serovar diversity and utilized phylogenetic approaches, along with proximal land use analyses, to identify relationships between environmental reservoirs and hosts. Across 19 sites, including animal agriculture, suburban, and forested areas, 10 L water samples were collected (*n* = 456), and cultured for *Salmonella*, followed by whole genome sequencing of isolates and deep serotyping of positive samples. Overall prevalence was 69% (314/456), and generalized linear mixed models showed that compared to proximal land use, seasonal weather patterns, including precipitation and humidity, significantly influenced recovery and complexity. Antimicrobial resistance was detected in 11% (33/314) of isolates, with 21% (7/33) classified as multidrug resistant. CRISPR-SeroSeq identified 37 serovars, and multiserovar populations were detected in 89% (229/258) of positive samples with sequencing data, averaging 3.7 serovars/sample. Comparison with national food animal production monitoring showed limited serovar overlap, with serovar Rubislaw dominating water samples but absent in agricultural data sets. Collectively, these results demonstrate extensive serovar diversity within *Salmonella* populations in freshwater systems, including clinically relevant serovars, and emphasize the need to develop a robust surveillance platform for source attribution and, ultimately, prevention of future outbreaks.

**IMPORTANCE:**

Contaminated surface water significantly contributes to global *Salmonella* illnesses, marking a critical need to assess serovars present and determine environmental variables affecting the population dynamics in this reservoir. We found that complex multiserovar populations, often including pathogenic serovars, occur in surface water regardless of proximal land use. Notably, many aquatic serovars are not detected in animal agriculture monitoring and the phylogeny presented here shows isolates are more closely related to human clinical than animal-source isolates. However, limited serotyping data are available for alternative reservoirs of foodborne illness, namely, wildlife, which hinders source attribution. This study highlights a significant gap in understanding environmental *Salmonella* transmission and underscores the importance of a One Health surveillance approach to protect public health.

## INTRODUCTION

Over one million deaths worldwide could be prevented by improving water safety measures and reducing the load of contaminating bacteria (1). *Salmonella enterica* is a key global cause of diarrheal illness and a global threat of antibiotic resistance, as serovar Typhi alone causes 9 million illnesses and 110,000 deaths via consumption of contaminated water (2). While typhoidal illness is uncommon in the United States, there are an estimated 1.35 million non-typhoidal illnesses each year, costing $4.1 billion (3, 4). Critically, 60%–80% of salmonellosis cases are not connected to a known outbreak, which underscores a critical lack of knowledge needed to prevent future illnesses (5). As an enteric organism, *Salmonella* outbreaks are often associated with consumption of contaminated meat and poultry, whose final products are routinely tested by the United States Department of Agriculture-Food Safety and Inspection Service (USDA-FSIS). Federal monitoring programs in meat and poultry are necessary to protect consumers, but it is also necessary to address the knowledge gap of *Salmonella* transmission as it pertains to wildlife and other potential environmental contributors associated with contamination in food production that are not surveyed. Notably, contaminated surface water is believed to be a significant contributor in disseminating foodborne pathogens as it may be used for crop irrigation and 44% of domestic *Salmonella* illnesses attributed to produce consumption (6–8). A historic serovar Saintpaul outbreak in peppers was linked to contaminated water, and, in 2024, a mixed serovar outbreak from cucumbers infected 551 individuals, and one outbreak strain was isolated from the agricultural water at the affected farms (8, 9). In another cucumber outbreak earlier this year, the outbreak strain was isolated from an environmental sample (10). It is likely that contaminated irrigation water contributes to many produce-related outbreaks, but traceback investigations do not often identify the exact sources due to the consumption or spoilage of products, along with the clearing of fields following harvesting. Because human clinical data, along with meat, poultry, and egg surveillance, comprise most publicly available data, any large-scale genomic attribution studies are skewed toward animal agriculture. Subsequently, food safety risk is not properly assessed for serovars arising from alternative sources, such as wildlife or contaminated water.

There are >2,600 *Salmonella* serovars, characterized by their combination of somatic and flagellar antigens (11, 12). Significant phenotypic differences occur across serovars, including host restriction, antimicrobial resistance, pathogenicity, and stress responses (13–16); this diversity may be further reflected in environmental reservoirs, as *Salmonella* may be found in water, soil, and a range of wildlife. For example, host-adapted serovar Choleraesuis is often recovered from wild boars and some subtypes of serovar Typhimurium are associated with wild birds (17–25). However, serovar associations with other wildlife, including mammals and reptiles, are largely understudied (26–34). *Salmonella* likely enters abiotic reservoirs such as water via fecal contamination from humans, wildlife, and animal agriculture and may be maintained in these environments following re-introduction or via persistence, as it has been shown to survive for at least 300 days in freshwater (35–43). Furthermore, as *Salmonella* often exists as mixed serovar populations, surface water may serve as a conduit for highly complex populations following contamination from multiple hosts (27, 44–47).

Conventional *Salmonella* isolation by culture-based methods can be quite sensitive; however, while selective enrichment media is necessary for *Salmonella* recovery, it can lead to skewed serovar population proportions (48). Furthermore, the number of colonies selected from a single petri dish can be limited. As an alternative approach, deep serotyping using CRISPR-SeroSeq (serotyping based on the sequences of native *Salmonella* CRISPR spacers) is an amplicon-based next generation sequencing approach that profiles the relative frequency of multiple *Salmonella* serovars in a single sample (49). Applying this method to *Salmonella*-positive surface water samples has shown that rivers harbor complex populations (46). At the time, this was attributed to the large size of the watershed (Susquehanna River, Pennsylvania) and that the land use in that region was multifaceted. The current study was designed to apply this methodology to gain an improved representation of the *Salmonella* population within a watershed, such that smaller creeks, close to the headwaters, were sampled, and to compare serovar differences between land that was selected for one primary use (i.e., human activity, animal agriculture, or national forest). Additionally, the previous study was conducted only during the spring and summer months, but meteorological variables, including temperature and precipitation, can influence *Salmonella* recovery from surface water, so the present study included year-round sampling (46, 50–54).

Here, we sought to assess the prevalence, population complexity, and antimicrobial resistance of non-typhoidal *Salmonella* within diverse freshwater environments representing four distinct creek systems in the southeastern United States. Additionally, we investigated the influence of proximal land-attribution and weather on *Salmonella* in these systems. Comparison of whole genome sequencing of isolates across the four creeks with publicly available data showed no overlap with isolates from animal agriculture. This, along with high *Salmonella* prevalence in a creek within a national forest, strongly suggests that wildlife contribute significantly to salmonellae found in water.

## RESULTS

### *Salmonella* prevalence and antimicrobial resistance

**Fig 1:**
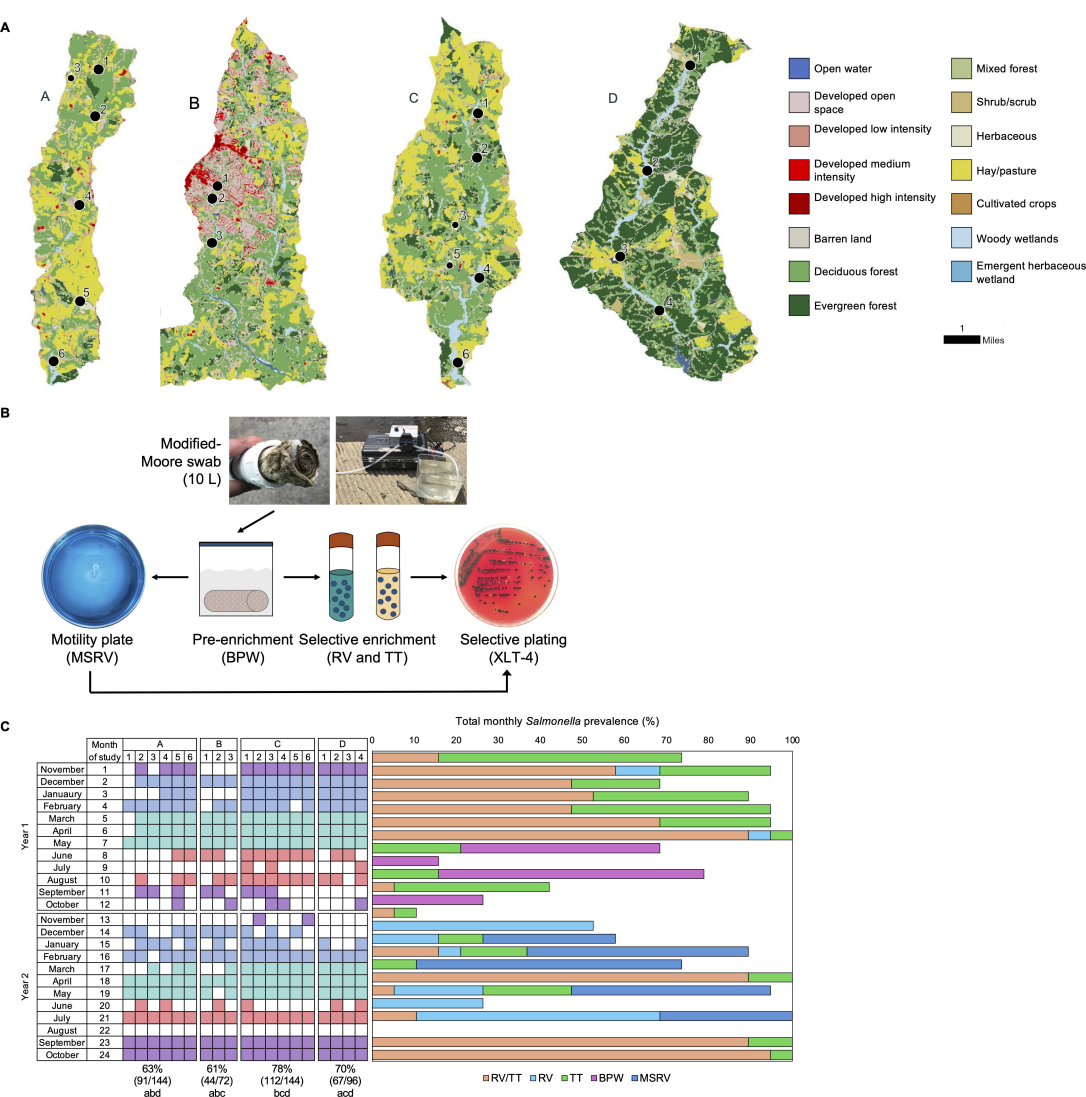
Sample collection and prevalence of *Salmonella* in watersheds. (**A**) Each watershed is shown with its corresponding land attribution from the national land cover database. Individual sites are shown as black circles with numbers representing each site. Tributary sites off the main creek stem are shown as smaller black circles. (**B**) Water samples were collected by pumping 10 liters through a modified Moore swab (MMS). The swab was returned to lab on ice and pre-enriched in buffered peptone water (BPW). Samples were transferred to selective enrichment media in parallel, including Rappaport-Vassiliadis (RV), Tetrathionate (TT), and modified semisolid RV (MSRV). Each enrichment was then plated onto an indicator plate, xylose lysine tergitol-4 (XLT-4) for confirmation. (**C**) Prevalence is shown with colored squares reflecting positive samples, and white squares are negative with overall sample number for each watershed shown at the bottom. The color of the square reflects the season of the sample collection, with observed differences in prevalence between them: fall (purple), winter (light blue), spring (light green), summer (light red) (Chi-squared test, *P* < 0.001). Additionally, *Salmonella* recovery varied by watershed, with relationships denoted below prevalence values (Chi-squared test, *P* < 0.05). Isolated colonies were saved from XLT-4 plates for all positive enrichments, including from BPW when RV and TT cultures were negative. MSRV was included in the protocol at the beginning of Year 2 due to sustained low prevalence in RV and TT starting in June of Year 1; however, isolates only from this media were collected in five out of 12 months in Year 2.

Over 24 months (November 2021–October 2023), 10 L samples was collected at 19 sites across 4 watersheds using modified Moore swabs (Fig. 1). Each watershed was selected for a land use, including animal agriculture (Watersheds A and C), a suburban community (B), and a national forest (D) (Fig. 1A). In total, 69% (314/456) of samples collected were *Salmonella*-positive (Fig. 1C). Prevalence differed across the watersheds (*P* < 0.05, Chi-squared Test) and was highest in Watershed C (78%; 112/144) and lowest in Watershed A (63%; 91/114); both systems were adjacent to animal agriculture. Prevalence also differed by season (*P* < 0.001), being highest in spring (93%; 106/114) and lowest in summer (47%; 54/114). Differences in *Salmonella* recovery were observed across culturing methods and fluctuated seasonally (Fig. 1C). In total, 11% (33/314) *Salmonella* isolates displayed AMR phenotypes, and 21% (7/33) of these were classified as multidrug resistant (MDR) (Fig. S1). The most common resistance was to streptomycin (29/33, 88%), and one isolate was resistant to seven antibiotics. These AMR isolates represented 15 serovars, as determined by whole genome sequencing, with 7 of human clinical importance as denoted by their presence in the top 15 serovars listed on the BEAM Dashboard from 2021 to 2023 hosted by the Centers for Disease Control and Prevention (CDC) (55).

### *Salmonella* populations in water

**Fig 2:**
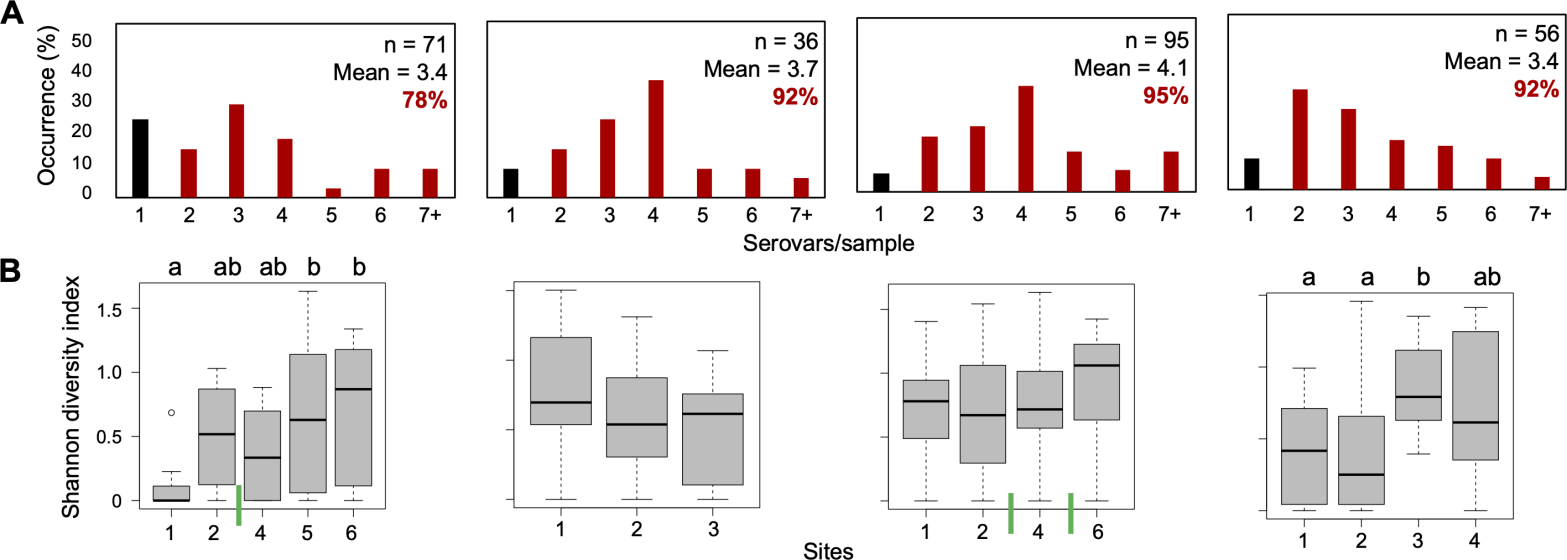
Multiserovar prevalence and diversity of watersheds. (**A**) Results of deep serotyping reveal the occurrence of multiserovar populations (red bar) compared to single-serovar populations (black bar). The number of positive samples, average serovars/sample, and percent of multiserovar populations for each watershed are included. (**B**) Shannon diversity indices of sites along the main stem of the creek, where lowercase letters show significant differences between sites using a Kruskal-Wallis and Dunn post-hoc test. The green vertical bars indicate where tributaries enter.

CRISPR-SeroSeq was applied to DNA isolated from broth cultures of all *Salmonella*-positive samples. A subset of these failed to amplify (*n* = 56), though the majority of these samples (45/56, 80%) were only positive in non-selective pre-enrichment media (buffered peptone water; BPW), which has been shown to be limited for CRISPR-SeroSeq analysis (56). Multiserovar populations were identified in 89% (229/258) of samples, with an average of 3.7 serovars per sample (range 1–13 serovars) and a total of 37 serovars detected (Fig. S3). On average, Watershed C had the highest number of serovars per sample (4.1) and the most samples containing multiple serovars (90/95, 95%), while Watershed A had the lowest (3.4; 55/71, 77%) (Fig. 2A). Concordant with *Salmonella* prevalence, the greatest number of multiserovar samples was detected in spring (81/86, 94%), with significant differences in complexity when comparing based on watershed (C to A), and season (spring to fall and winter) (*P* < 0.05, Kruskal-Wallis test with Dunn post-hoc). Additionally, the increase in Shannon diversity indices of *Salmonella* populations across sites following the main stem of each creek showed that serovar complexity increased in downstream collection sites within watersheds A and D (Fig. 2B). We did not note differences in serovar complexity following a tributary joining the main stem (Watersheds A and C, indicated by green bars in Fig. 2B).

### Models to assess contribution of weather and land attribution

A generalized linear mixed model (GLMM) was used to analyze weather and land attribution data, including non-linear and interaction effects between and among variables, to determine the likelihood of *Salmonella* detection (Table S1). The models used fall as the baseline season (intercept). Based on odds ratio (OR) calculations with 95% confidence intervals (CI), the likelihood of *Salmonella* detection was higher in spring (*P* < 0.05, OR = 2.09, CI 1.26–3.48) and lower in summer (*P* < 0.05, OR = 0.06, CI 0.04–0.11). For serovar complexity, spring was also a positive predictor of higher complexity (*P* < 0.05, OR = 2.10, CI 1.16–3.79) (Table S2). There were other significant variables (e.g., wind speed, precipitation), but the OR values did not indicate strong relationships. The relationship between weather variables showed a strong association between maximum temperature and minimum temperature (*r* = 0.89), along with radiation (*r* = 0.75) (Fig. S2).

### Distribution of serovars across watersheds

**Fig 3:**
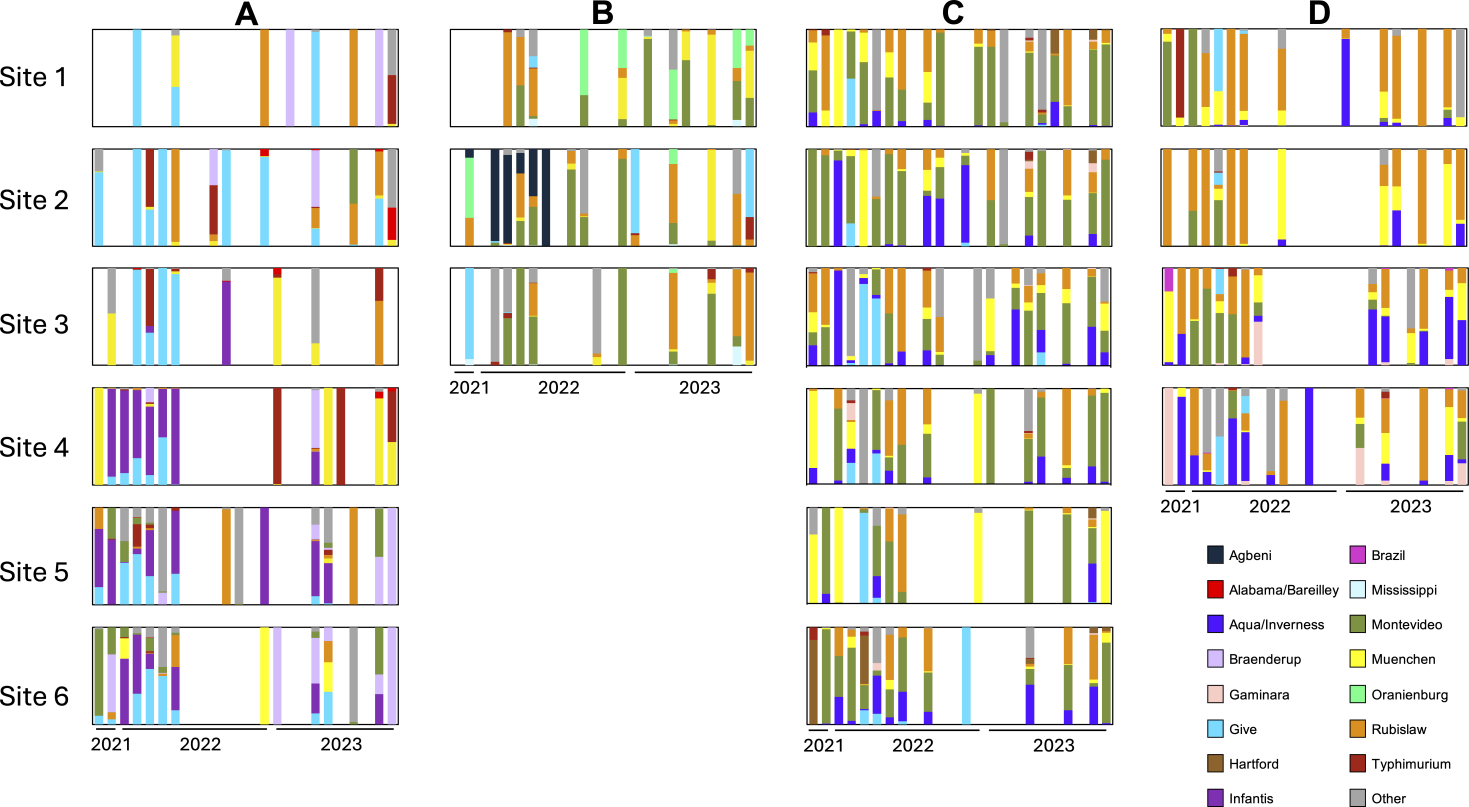
Top eight serovars present in each watershed. Deep serotyping results are organized by site (sites 1–6) and watershed (**A–D**). Each box represents the 24 months across a site, where colored bars show the proportion of each serovar present in that sample. Months with no bar represent a negative sample or a sample where deep serotyping was unsuccessful. Serovars outside of the top eight are classified as “other” and represented by a gray bar. The suffixes (-I, -II, -III) for some serovars refer to polyphyletic lineages.

When comparing all serovars detected from at least five samples across the whole data set, serovars Give I, Muenchen I, Rubislaw, and Typhimurium were shared among all watersheds (Fig. S4A). Alternatively, some serovars were unique to a watershed, such as serovars Agbeni and Oranienburg in Watershed B and Anatum in Watershed C. All serovars identified in over five samples from Watershed D were non-unique. Watershed A contained the most serovars (*n* = 30), while Watershed D contained the least (*n* = 18). Principal coordinate analysis based on Jaccard distance showed that *Salmonella* populations were most consistent in Watershed A (Fig. S4B). The most common serovar in watersheds A, B, C, and D was Give I (*n* = 32), Rubislaw (*n* = 23), Montevideo II (*n* = 72), and Rubislaw (*n* = 46), respectively, and their relative abundances differed over time and within watersheds (Fig. 3). For example, serovar Infantis was almost exclusively isolated from sites 4–6 in Watershed A. In Watershed C, serovar Agbeni was only found at site 2 in the first 6 months of *Salmonella*-positive samples.

### Genomic comparison of study serovars with publicly available genomes

**Fig 4:**
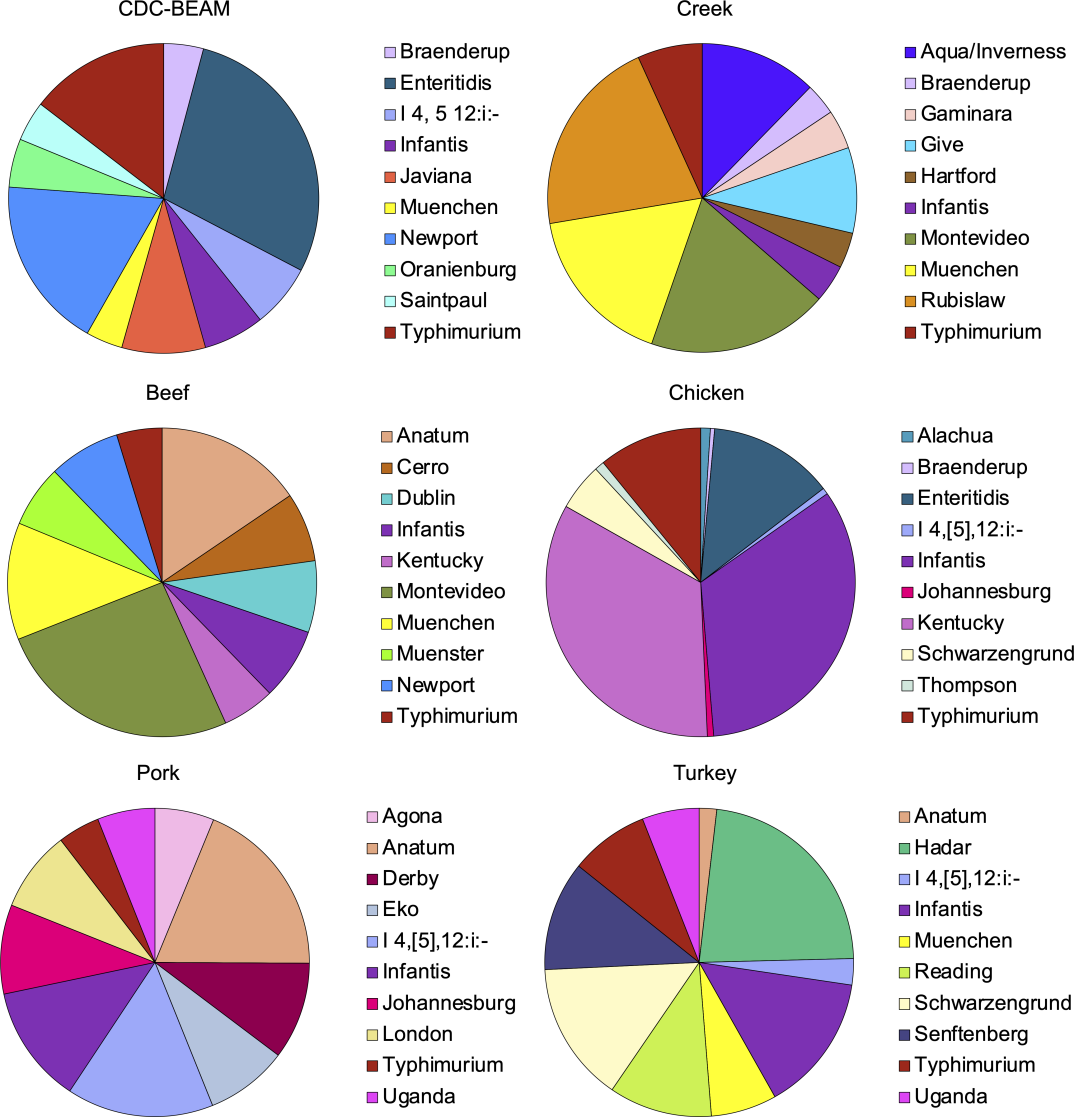
*Salmonella* serovars differ between animal agriculture, surface water, and clinical cases. The top 10 most common serovars for each food animal monitored by the USDA-FSIS, clinical cases reported to the CDC, and creek samples from this study are displayed. Serovar data for the commodity groups were accessed from the publicly available USDA-FSIS sampling repository (USDA-FSIS, 2024) and included samples that were collected between November 2021– October 2023. Clinical isolates were accessed through the CDC BEAM Dashboard (CDC, 2024) and included cases for the entirety of 2021–2023. Polyphyletic serovars are not delineated by the USDA-FSIS or CDC, so the suffixes were removed from serovars identified by deep serotyping and both results from both Montevideo lineages were combined for the creek pie chart. Serovar Typhimurium and its monophasic variant, I 4, [5], 12:i:-, cannot be distinguished by the CRISPR spacers.

**Fig 5:**
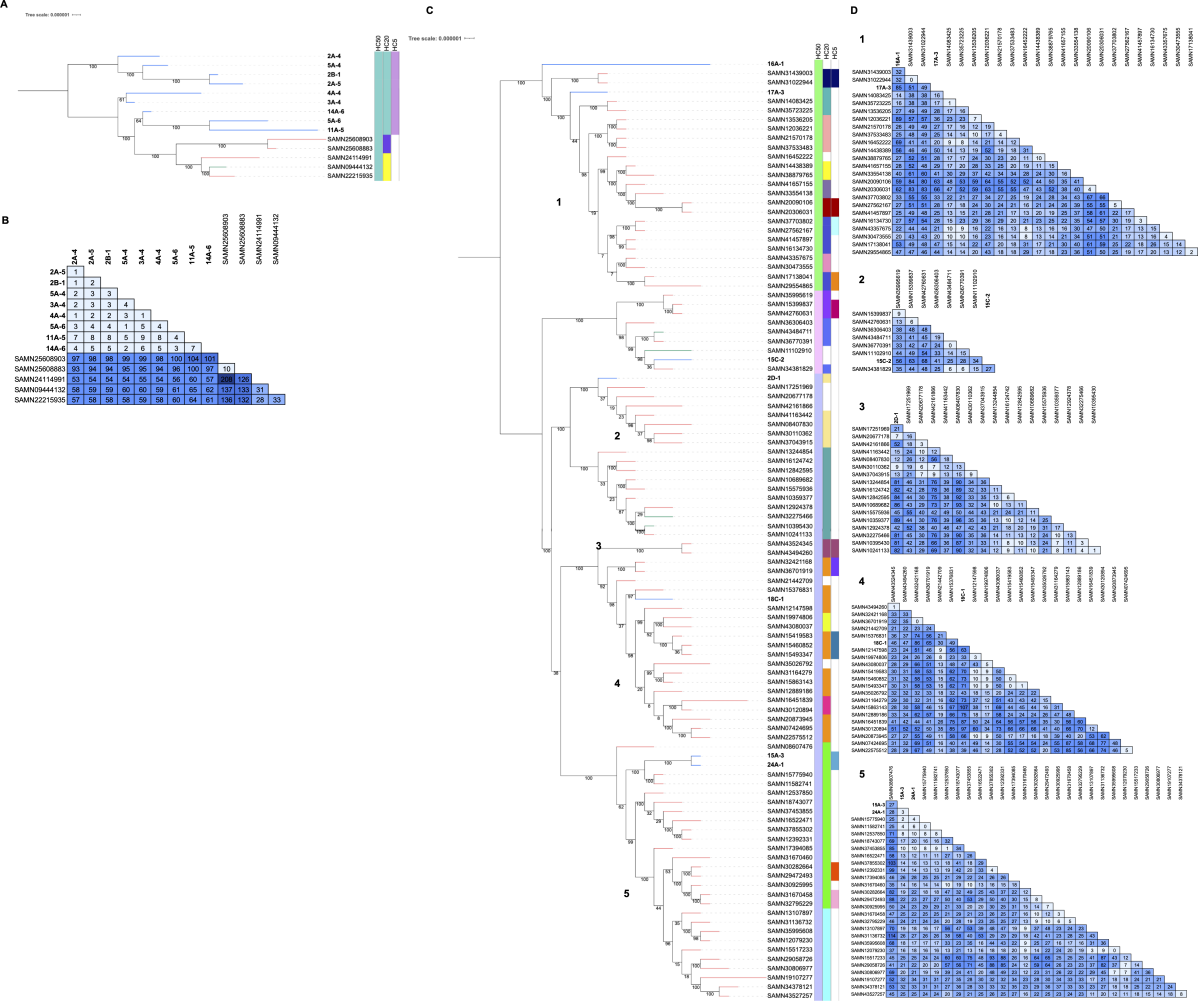
Serovar Infantis and Typhimurium isolates collected in this study are not closely related to other isolates from mixed environmental sources. Study isolates are bolded, with the label format of month-watershed-site, and NCBI isolates are listed by sample ID with branch color corresponding to isolation type (human clinical: red, environmental/other: green, creek: blue). Isolation type was provided by NCBI metadata, and the HierCC schemes were determined through Enterobase. Schemes were only reported if they are shared between two or more isolates. The phylogenies are rooted at the midpoint and include all isolates within the two SNP clusters most closely related to study isolates. HC20 and HC5 values were added to reflect that the study isolates were greater than 20 and 5 allelic differences from the NCBI isolates, respectively. For the matrices, darker shading indicates a greater SNP distance. (**A**) Serovar Infantis core genome phylogeny, (**B**) Serovar Infantis SNP matrix, (**C**) Serovar Typhimurium core genome phylogeny, (**D**) Serovar Typhimurium SNP matrix based on corresponding phylogenetic clades (1–5).

The USDA-FSIS monitors meat and poultry products, including beef, chicken, pork, and turkey. Based on regulatory sampling data sets from 2016 to present (with the exception of pork which only included data sets starting in 2019), the top 10 serovars by prevalence were identified for each commodity group and compared to serovars we found in our water samples (Fig. 4). The most commonly identified serovars for turkey, chicken, pork, and beef were Reading (24%), Kentucky (33%), Anatum (18%), and Montevideo (23%), respectively. Alternatively, serovar Rubislaw was most often found in creek samples (22%), which was one of several serovars absent from the animal agriculture data, including Aqua/Inverness, Gaminara, Give I, Hartford, and Mississippi II. Serovars Infantis and Typhimurium were the only serovars collected from this study that were also routinely identified in all four primary domestic food animals. Therefore, we completed a phylogenetic comparison of the study isolates belonging to these two serovars against publicly available genomes in the National Center for Biotechnology Information (NCBI) Pathogen Detection system, which hosts human clinical and USDA-FSIS whole genome sequences, among others (Fig. 5; Fig. S5 and S6; Tables S4 to S9). Notably, hierarchical clustering based on the core genomes determined that there were more than 20 and 50 allelic differences between serovar Infantis and Typhimurium study isolates and the most closely related isolates, respectively (Fig. 5). Alternatively, the study isolates were most closely related as they were all within five or fewer allelic differences (HC5). For serovar Infantis, four of the five most closely related genomes were from human clinical isolates and the other was from a market swine in the midwest (Table S4). Importantly, the phylogenetic analyses (Fig. 5A) showed that these five most closely related genomes did not share an HC20 type with the creek genomes, indicating that the creek isolates are genetically quite distinct from those in NCBI. Additional analysis using the CFSAN SNP Pipeline revealed that these creek isolates differ by 53–104 SNPs from the NCBI isolates (Fig. 5B). Similarly, for serovar Typhimurium, 91/95 of the most closely related isolates were from human clinical cases, and the remaining four were environmental, collected from almonds, white-tailed deer and emu feces, and a dairy cow from the northeast (Fig. 5C; Table S7). There was more variation in the SNP differences between the serovar Typhimurium creek isolates and NCBI isolates (Fig. 5D), but only a select number of the human clinical isolates (*n* = 6) were within 10 SNPs of the study isolates, and these were shared between two SNP clusters, as defined by NCBI.

## DISCUSSION

Each day, 198 billion gallons of surface water are used in the United States with 61 billion gallons used for irrigation alone (57). Therefore, it is pertinent to investigate *Salmonella* in surface water. Our data show that the *Salmonella* strains recovered do not match to food animal isolates, suggesting alternative reservoirs. Specifically, there is a need to understand the contribution of wildlife to *Salmonella* populations within freshwater ecosystems, as these hosts may be introducing pathogenic serovars and contributing to the observed AMR levels (32, 58–64). Applying a One Health approach is necessary to mitigate the impacts of environmental *Salmonella* since there is opportunity for cyclical transmission between animals and humans via shared environmental reservoirs, such as water.

As surface water is commonly used for agricultural water (i.e., irrigation), the Food Safety Modernization Act-Produce Safety Rule (FSMA-PSR) details its impact on food safety risks and provides guidance on best practices (FSMA Final Rule on Produce Safety, FDA 2024). In-field, antimicrobial treatments of irrigation water have limitations since not all growers have access to the necessary resources and farm size can greatly impact the efficacy of these treatments (65–68). The extensive application of surface water and variability of sources also complicates risk assessment as *Salmonella* testing can be lengthy and costly, and there is likely not a one-size-fits-all control strategy. Many previous studies have sought to find an indicator organism that is easier to identify in the field and suggests the presence of *Salmonella* contamination. While *E. coli* has been considered an indicator of *Salmonella*, due to its association with fecal contamination and niche as enteric bacteria, some work has highlighted inconsistencies in the applicability of this organism (51, 69, 70).

Overall *Salmonella* prevalence in this study was 69% (314/456), which is comparable to other studies in this region (50, 51, 71, 72). Notably, while we observed seasonal trends, we also found high variability in prevalence. For example, the monthly incidence shifted from 100% (19/19) of sites to 0% (0/19), and back to 100% (19/19) over just 3 months during 2023. Reduced rain and increased temperature during the summer months could contribute to reduced nutrient availability and *Salmonella* influx to the environment. Previous work has shown lower *Salmonella* prevalence in summer, while others show higher prevalence during summer, suggesting variability across different watersheds. Similarly, precipitation has been associated with *Salmonella* in some studies but not others (40, 46, 50–53, 73). Developing predictive models for the likelihood of *Salmonella* detection in the environment is a complex problem due to the abundance of external interactions and present bacterial populations, along with regional differences in weather, watershed land-use composition, and biochemical properties in water, including nutrient availability (51, 69, 74–77). A potential caveat to this work was the use of weather stations to assess precipitation; this was a logistic decision as we had 19 sites to cover. Since “pop-up” storms can be common in the southeast in the summer and can be highly localized, use of meters close to sampling sites may have been more helpful in assessing precipitation levels and, thus, addressing run-off impacts. Furthermore, a model is only as good as the input data so cultural limitations of *Salmonella* identification and characterization, as well as variance in sample collection methods, may impact model development. One recent study demonstrated the importance of detection methods as both *Salmonella* prevalence and types differed in water samples that were processed with separate approaches (78). Another study showed that triplicate samples collected from the same location at the same time can also result in different *Salmonella* outcomes (79). A potential alternative to cultural isolation is the use of bacteriophage as sensors for *Salmonella* in water, as has been applied for serovar Typhi (80). In the absence of a standardized method for *Salmonella* isolation from water, this will continue to be a caveat. We suspect that by profiling the serotype population more fully by deep serotyping, we were able to mitigate this, at least in part.

While the GLMM approach used in this study identified significant predictors for both *Salmonella* prevalence and serovar complexity, the relationships were not strong (OR < 2.1); this highlights the complexity of determining the primary contributing factors to *Salmonella* in water and suggests that there may be multiple variables cumulatively influencing *Salmonella* contamination in water. We did not measure any water parameters in this study (e.g., water temperature, dissolved oxygen, pH), as direct access to the creeks was limited in some instances. However, previous work has demonstrated that the relationship between turbidity levels and bacterial populations in water is dynamic (51, 52, 81), which further emphasizes that there is a complex network of biotic and abiotic factors that contributes to *Salmonella* abundance in water and there is not likely one main driver. Furthermore, the sampling sites do not exist within a vacuum, and this is one of the limitations in modeling; for example, there may be human activity, such as leaking or malfunctioning septic tanks, affecting *Salmonella* populations in the watershed located within agricultural areas.

In this study, 11% of isolates displayed AMR, which is slightly greater than a recent study performed in Georgia where isolates collected from water in the Middle Oconee River contained AMR phenotypes in 4.4% of samples (72). Our results indicate that antimicrobial resistance genes are circulating even when human impact is limited but wildlife may be abundant, such as in the case of samples collected in a national forest (Watershed D). The presence of MDR in these isolates raises concern over the dissemination of antimicrobial resistance genes (ARGs) via various pathways, including direct and indirect wildlife interactions, horizonal gene transfer from bacterial communities, or human activity. As surveillance of wildlife has remained limited, an accurate assessment of the prevalence of both foodborne pathogens and ARGs is currently not within reach (and is a limitation of the current study), but this information can be supplemented with routine watershed sampling. Otherwise, this limitation will continue to be a barrier to effective risk assessment as wildlife interaction in agricultural water and produce fields is a multifaceted problem with complex solutions (82). Future studies that pair water sampling with wildlife sampling would help to address this gap, especially for salmonellae known to circulate in wildlife (e.g., some serovar Typhimurium strains in wild birds and serovar Choleraesuis in wild boars). It is possible that wildlife migration over different times of the year may explain the seasonal differences that we observed. Again, this would require identifying wildlife to then assess proximity to water sources during migration.

When this study began, two different selective enrichment media, Rappaport-Vassiliadis (RV) and tetrathionate (TT) broths, were utilized and enrichments were plated onto xylose lysine tergitol-4 (XLT-4) agar. In the first 12 months, if *Salmonella* was not recovered following TT or RV enrichment, the pre-enrichment (BPW) culture was plated directly onto XLT-4 and, in some cases, yielded culturable isolates. The limited recovery may be indicative of the stress upon cells in an aquatic environment, from which the non-selective enrichment for 24 h prior was not sufficient for full recovery; thus, salmonellae were not able to proliferate in the harsh environment generated by either RV or TT. This has important implications for routine environmental *Salmonella* monitoring and subsequent public health responses as stressed or starved bacteria may enter a viable but nonculturable state and evade conventional detection methods (83). This limited recovery of *Salmonella* in some samples may also explain why many of the libraries that failed to provide deep serotyping data came from BPW cultures. Twelve of the failed libraries from BPW generated some serovar information but were below our threshold of 1,000 reads, therefore not included in our anaylsis. We suspect that for many of the failed libraries, the *Salmonella* level was low and/or there were high levels of background microbes that can also grow in BPW (e.g., *E. coli* whose CRISPRs are also targeted by our primers) and, thus, did not result in good sequencing libraries for *Salmonella*.

Surface water harbors complex mixed-serovar *Salmonella* populations, with an average of three serovars per sample identified in a large river (range: 1–10) (84). Findings here show comparable, if not higher complexity in small creeks (average of 3.7 serovars per sample, range 1–13). This observation is significant as it demonstrates that these complex populations can form close to creek headwaters and are relevant to food safety. A previous study utilizing 16S analyses showed higher microbial community complexity upstream, with populations becoming more stable and less complex further downstream in watersheds (85, 86). Focusing on a single species, the *Salmonella* data presented here are inverse; in two creeks (A and D), the complexity of serovar populations increased downstream, while creeks B and C showed no differences (Fig. 2B). A potential explanation for increasing complexity downstream is that the salmonellae are moving in the creek stem, and those populations are augmented by additional salmonellae that enter at later points, collectively accumulating downstream. Additionally, *Salmonella* can invade and live in amoebas, ciliates, and biofilms which promote long-term survival in aquatic environments (36, 38, 41, 43). Nonetheless, our results demonstrate that most samples contained multiserovar populations, which further emphasizes that selecting multiple colonies is necessary to reflect the full *Salmonella* profile within each sample (87).

The *Salmonella* populations identified here in surface water are more complex than those observed in food animals or in wild birds (88–92); this is likely due to surface water acting as a catch-all for salmonellae from multiple different sources. Additional research is needed to identify the factors that influence *Salmonella* complexity within all three components of a One Health scheme: animals, humans, and the environment. Furthermore, the expansion of environmental surveillance could help to more quickly identify outbreak strains and complete epidemiological traceback, as it would increase the depth of publicly available *Salmonella* genomes. Subsequently, any generated whole genome sequences could provide new genomic insights on *Salmonella* transmission, including virulence factors needed for host colonization, environmental persistence, or stress response (93). Previous work has demonstrated the utility of environmental surveillance approaches such as wastewater testing to measure the burden of *Salmonella* illness or identify cases that would otherwise go unreported (94–98). Since salmonellosis is often self-limiting within 7–10 days, it is significantly underrepresented in clinical cases, with an estimated 39 cases not reported for each lab-confirmed case, so wastewater monitoring may aid in understanding the true transmission and infection dynamics.

Human salmonellosis is often attributed, directly or indirectly, to food animal production via improper storage or consumption of contaminated meat. While some serovars that are often found in food animals were also found in our study (e.g., Typhimurium, Infantis, Braenderup, Muenchen, Montevideo), the watersheds were dominated by serovars that are not typical of these production systems. Serovars Rubislaw (Watersheds B–D), Give (Watershed A), and Aqua/Inverness (Watersheds A, C, D) have been commonly associated with surface water environments, with reptiles as a reservoir for serovar Rubislaw, occasionally causing human salmonellosis, but animal reservoirs are not often identified for the latter two serovars (45, 50, 72, 99–102). There are some suggestions that serovar Give is linked to cattle (103, 104), but it this serovar is not frequently found by USDA-FSIS inspection of beef facilities. Notably, in the state of Georgia, serovar Rubislaw (*n* = 174) accounts for almost one-tenth of the illnesses caused by serovar Enteritidis (*n* = 1,956), which is the leading serovar in human cases, demonstrating its ability to become systemic and cause serious illness (55). Of these cases, nearly 13% come from blood or urine (rather than from stool), suggesting this serovar causes serious illness. The current study did not include source attribution of fecal contamination within these watersheds, but phylogenetic analyses suggested that the collected serovar Infantis and Typhimurium isolates did not originate from food animal production. Importantly, the serovar Infantis isolates from this study are not related to the clonal lineage responsible for the recent *Salmonella* outbreak in raw chicken products, as determined by genomic comparisons and the lack of pESI megaplasmid within the sequenced isolates (105, 106). While further studies are needed to attribute serovars within these highly variable aquatic environments to likely hosts, the surveillance framework developed in this study can be helpful in identifying the salmonellae that are being circulated in respective environments.

In conclusion, this study highlights the complexity and variability of *Salmonella* populations within watersheds influenced by diverse land uses and climatic factors. Our findings demonstrate that multiserovar populations are prevalent within surface water and suggest that environmental factors such as precipitation and humidity significantly influence *Salmonella* prevalence and complexity although it is a multifaceted relationship. Population analyses showed increasing complexity further downstream, as well as higher serovar complexity in the spring compared to fall and winter. The identification of 37 serovars, including those of human concern harboring antimicrobial resistance, highlights the need for enhanced surveillance strategies that integrate environmental and wildlife monitoring. In particular, our serotyping data and subsequent whole genome sequence analysis demonstrated that there is a significant discordance between environmental, clinical, and animal agriculture isolates. Current *Salmonella* surveillance data are dominated by those made available by federal agencies such as the U.S. Food and Drug Administration, CDC, and USDA-FSIS. Under a One Health umbrella, understanding *Salmonella* in the environment and in wildlife is considerably understudied, and there is a critical need to close this loop. Future work should prioritize understanding the transmission pathways between these reservoirs and improving predictive modeling to inform targeted interventions and public health policies.

## MATERIALS AND METHODS

### Sample site selection

Four distinct watersheds in the southeastern United States were selected based on their surrounding land attribution, including animal agriculture, national forest, and suburban communities (Fig. 1A). The number of sites within each watershed varied, based on size of the watershed and accessibility of sites from the road. For each watershed, at least one sample was collected from within 0.5 miles of the headwater. All sampling locations were within first or second order channels and were upstream of any sewage or drinking water treatment facilities.

### Sample collection and *Salmonella* isolation

A modified Moore swab and peristaltic pump were used to collect a single sample of 10 L of creek water from each site (*n* = 456 samples) (Fig. 1B) (107). Modified Moore swabs were placed at approximately midchannel resting on the sediment for the duration of pumping. The swabs were stored on ice until returning to the lab, where 100 mL of buffered peptone water (BPW; Neogen, Lansing, MI) and novobiocin (40 mg/L; Thermo Scientific Chemicals, Waltham, MA) were added. The swabs were hand-massaged for 1 min and then incubated shaking at 42°C for 20–24 h. Following incubation, 1 and 0.1 mL of BPW was transferred into 10 mL of tetrathionate (TT; Hardy Diagnostics, Santa Maria, CA) and Rappaport-Vassiliadis (RV; Hardy Diagnostics, Santa Maria, CA) broth, respectively, and statically incubated at 37°C for 20–24 h. Starting at month 13, 0.1 mL of enriched BPW was inoculated into a modified semisolid Rappaport-Vassiliadis (MSRV; Hardy Diagnostics, Santa Maria, CA) plate and incubated at 37°C for 20–24 h. *Salmonella* presence was tested by streaking the TT and RV cultures, along with any presumptive *Salmonella* growth from the MSRV plates, onto xylose lysine tergitol-4 (XLT-4; Hardy Diagnostics, Santa Maria, CA) plates, followed by incubation at 37°C for 24–48 h. During the first 12 months, if the TT and RV enrichments did not result in presumptive *Salmonella* colonies, then the enriched BPW culture was directly plated onto XLT-4 (Fig. S1B). Three colonies were selected and restreaked for isolation onto new XLT-4 plates. After incubation at 37°C for 24–48 hours, a single colony was streaked onto Luria-Bertani (LB; Hardy Diagnostics, Santa Maria, CA) agar and then confirmed with serum agglutination (BD Difco, Franklin Lakes, NJ). All confirmed *Salmonella* isolates were saved via glycerol stocks in a −80°C freezer. From all enrichments, 1 mL was removed and centrifuged at 18,000 × *g* for 3 min, then the supernatant was removed, and the pellets were stored at −20°C for later use.

### *Salmonella* population analysis

To identify the populations of *Salmonella* within water samples, enrichments resulting in *Salmonella* culture positive samples were processed individually by centrifuging 1  mL of each positive selective enrichment at 18,000 × *g* for 3 min. Total genomic DNA was isolated from the resulting pellet using a Promega Genome Wizard kit (Madison, WI) according to the manufacturer’s instructions and resuspended in 200 µL of molecular-grade water. A total of 2 µL of this template was used in the PCR for CRISPR-SeroSeq with primers targeting the conserved direct repeat sequences within *Salmonella* CRISPR arrays (49, 88). Primers also included index sequences (using the Illumina Nextera index sequences) which facilitated multiplexed, high throughput sequencing. PCR products were purified using the AMPure system (Beckman Coulter, Indianapolis, IN) and pooled in approximate equimolar ratios. Pooled libraries were sequenced using the Illumina NextSeq 550 platform (Illumina, San Diego, CA) mid output 150 cycle v2.5 kit with single-end reads. A water negative-control and a positive control containing *Salmonella* serovar Enteritidis genomic DNA with a known CRISPR profile were included in the library. Sequence reads were parsed and matched in a local BLAST search to a lab-curated database of over 160 serovars (88), and the results were output as Excel files. Serovars were called only if they contained multiple CRISPR spacers that were unique to that serovar. Where there were sufficient *Salmonella* sequence reads (>1,000 reads) for both the TT and RV enrichments, the relative frequency of each serovar was normalized using DESeq2 (108) across both enrichments to provide a single serovar profile. Stacked bar charts and heat maps were generated in Excel. Within this data set, serovars Alabama and Bareilly III, Aqua and Inverness, and Johannesburg and Urbana are unable to be distinguished based on CRISPR spacer content, respectively. Raw sequence reads were uploaded to NCBI (PRJNA1252771).

### Calculating land attribution for each sampling site

Land cover data were obtained from USGS National Land Cover Database (https://www.usgs.gov/centers/eros/science/national-land-cover-database). Watershed data were obtained from U.S. Geological Survey national hydrography database (https://www.usgs.gov/national-hydrography/access-national-hydrography-products). Inverse-distance weights (IDW) were used to characterize land cover within watersheds to give greater weight to land closer to the sampling site (109). The IDW proportions for each land cover class were calculated for the following distance intervals (m): 0–100, 100–250, 250–500, 500–1,000, 1,000–2,000, and 2,000–5,000 upstream of the sampling sites. Land cover data for each watershed was extracted using ArcGIS Pro version 3.1 (https://www.mrlc.gov/data/legends/national-land-cover-database-class-legend-and-description).

### Weather data collection

Meteorological data were collected using three weather stations that were closest to the four creeks. Measurements included maximum and minimum air temperature, relative humidity, average wind speed, solar radiation, and total precipitation; these were measured daily between 12:15 am and 11:59 pm. Data were collected for 2 days preceding sampling and the day of sampling and used in the final models. Multicolinearity was evaluated prior to modeling using variance inflation factors (VIF < 4), which led to the exclusion of highly correlated variables such as radiation. All weather predictors represented conditions averaged across the sampling day and the two preceding days, except precipitation, which was modeled as the cumulative total over the same period. Lagged predictors beyond this were not incorporated to limit model complexity.

### Predictive modeling for *Salmonella* prevalence and complexity

Preliminary exploratory data analysis was performed to determine the modeling input. Analysis of variance inflation factors (VIFs) was used to assess the multicollinearity among the weather and land attribution variables, with a cutoff VIF value of 4. Additionally, all land attributes variables which comprised less than 5% of the total land cover per watershed were removed. Generalized linear mixed models (GLMMs) were developed using lme4 package in R (https://cran.r-project.org/web/packages/lme4/index.html) to investigate how the weather, land attribution, and temporal factors affected the likelihood of *Salmonella* detection and population complexity (number of serovars per sample). In these models, all variables were considered fixed effects, while the watershed was included as a random effect to account for pseudo-replication. The final GLMM was chosen following forward selection to determine if any quadratic or interaction effects should be added, along with backward selection to remove any insignificant variables from the model.

### Statistical testing

All analyses were performed in R version 4.3.3. PCoA was performed using the Vegan package in R (https://cran.r-project.org/web/packages/vegan/). The deep serotyping results were normalized using the DESeq2 package (http://www.bioconductor.org/packages/release/bioc/html/DESeq2.html) to adjust the read counts per sample based on the size factors present (108, 110).

### Antimicrobial susceptibility testing

One *Salmonella*-positive isolate from each site in each month was selected for antimicrobial susceptibility testing. When selecting isolates, preference was given to the first colony recovered from TT as this provided the highest recovery (Fig. 1C), followed by RV and BPW. To measure the susceptibility of the *Salmonella* isolates, the Kirby-Bauer disk diffusion assay was used to test 10 different antibiotics: ampicillin (10 µg), amoxicillin-clavulanic acid (20/10 µg), ceftriaxone (30 µg), gentamicin (10 µg), streptomycin (10 µg), tetracycline (30 µg), ciprofloxacin (5 µg), sulfamethoxazole-trimethoprim (25 µg), nalidixic acid (30 µg), and chloramphenicol (30 µg) (BD Diagnostics, Franklin Lakes, NJ). Bacterial overnight cultures were standardized to 0.5 McFarland (ThermoScientific Remel, Lenexa, KS) and spread plated onto Mueller-Hinton agar (BD BBL, Franklin Lakes, NJ). Antibiotic disks were placed on the plates, followed by incubation for 18–22 h at 37°C. Zones of inhibition were measured with a ruler, and the results were interpreted with the CSLI standards to determine if isolates were susceptible, intermediate, or resistant (111). Isolates were categorized as multidrug resistant if they were resistant to three or more classes of antibiotics. For subsequent analyses, an isolate with intermediate resistance was considered “susceptible.”

### Whole genome sequencing

To confirm the serovar of each isolate with antimicrobial resistance and compare the relatedness to publicly available *Salmonella* genomes, a single colony was picked from the LB plate used for *Salmonella* confirmation, inoculated in an LB agar stab, and shipped to the United States—Food and Drug Administration, Center for Food Safety and Applied Nutrition (FDA-CFSAN, now Human Food Program) for library preparation and sequencing. Isolates were reconfirmed as *Salmonella* with VitekMS (Biomerieux, Marcy-l'Étoile, France). Libraries were prepared with the Illumina DNA Prep kit (Illumina, San Diego, CA, USA) on the Sciclone G3 NGSx iQ Workstation (Perkin Elmer, MA, USA). Sequencing was done on the Illumina NextSeq 2000 using the NextSeq 1000/2000 P2 reagents 300 cycles with the 150 paired-end chemistry (Illumina). Raw Illumina sequence reads were uploaded to NCBI (BioProject PRJNA292661, PRJNA292661, PRJNA292663) for further processing through the Prokaryotic Genome Annotation Pipeline and the resulting assemblies generated on NCBI were used for further analysis.

### Phylogenetic analyses

Due to the overlap of serovars Infantis and Typhimurium in both water and food animal sources, we compared the study isolates against publicly available genomes. For these phylogenetic analyses, we used iterative steps to reduce computational demands and to increase the resolution of relatedness in the final tree. Metadata, including accession numbers, for all isolates with computed (sero)type matching serovar Infantis or Typhimurium were downloaded from NCBI Pathogen Detection on 10 October 2024 (Table S6 and S7). The following steps were completed in parallel for both serovars of interest. To reduce selection bias, the first isolate with an assembly was chosen as the representative for each single nucleotide polymorphism (SNP) cluster, as organized on Pathogen Detection. Isolates within a SNP cluster are considered highly related (112). Some SNP clusters were excluded from further analyses due to isolates lacking a GenBank assembly accession, for a total of 650 and 3,057 representative genomes/SNP clusters of serovars Infantis and Typhimurium, respectively. For each serovar, a core genome alignment was generated with the NCBI and creek genomes using Roary v3.13.0 (113) with the default settings. The resulting alignment, consisting of 3,124 and 3,511 genes for serovars Infantis and Typhimurium, respectively, was used to generate a phylogenetic tree with Very Fast Tree (114) with the default settings. All trees were visualized with iTOL (115). To increase the phylogenetic resolution, the clades containing the study isolates and all representative isolates within two most recent common ancestors were selected to generate a core genome alignment (yellow highlight, Tables S5 and S8; Fig. S5 and S6). As the decreased number of isolates allowed for more computationally intensive algorithms, RAxML-NG v1.2.2 (116) (1,000 bootstrap replicates, GTR + G model with default settings) was used to infer a maximum-likelihood phylogeny for the subset clades. For this smaller subset, we re-assembled the genomes using SPAdes v3.15.5 with the default settings (117). Based on the resulting tree, we determined which two SNP clusters contained the most closely related for each creek isolate (Infantis: *n* = 2 SNP clusters; Typhimurium: *n* = 14 SNP clusters) (blue highlight, Tables S6 to S9; Fig. S5 and S6) and analyzed all annotated assemblies within those clusters (Infantis: *n* = 5 isolates; Typhimurium: *n* = 95 isolates) to create a final phylogeny of the most closely related isolates on NCBI to the study isolates, using roary, RAxML-NG, and iTol as described above. To determine the hierarchical clustering scheme (HierCC) for all isolates included in the final phylogeny, the corresponding raw reads were transferred from NCBI Short Read Archive to Enterobase (118–120). The HierCC 50, 20, and 5 categories were included to demonstrate the genetic distance between study isolates and NCBI isolates based on the clusters calculated with the distance between core genome multilocus sequence types. Additionally, the CFSAN SNP Pipeline was used to generate a pairwise comparison matrix of each isolate included in the final phylogenies (120).

## Data Availability

The Illumina reads for whole genome sequencing (PRJNA186035, PRJNA292661, PRJNA292663) and for CRISPR-SeroSeq (PRJNA1252771) are available on NCBI.

## SUPPLEMENTAL MATERIAL

Supplemental figures aem.01770-25-s0001.pdf Figures S1 to S6.

Fig. S3 aem.01770-25-s0002.xlsx Heatmap of deep serotyping data.

Supplemental tables aem.01770-25-s0003.xlsx Tables S1 to S4, S6, S7, and S9.

Table S5 aem.01770-25-s0004.xlsx Select metadata downloaded from NCBI for serovar Infantis isolates included in Fig. S6A.

Table S8 aem.01770-25-s0005.xlsx Select metadata downloaded from NCBI for serovar Infantis isolates included in Fig. S7A.

ASM does not own the copyrights to Supplemental Material that may be linked to, or accessed through, an article. The authors have granted ASM a non-exclusive, world-wide license to publish the Supplemental Material files. Please contact the corresponding author directly for reuse.

## References

[1] Wolf J, Johnston RB, Ambelu A, Arnold BF, Bain R, Brauer M, Brown J, Caruso BA, Clasen T, Colford JM Jr, Mills JE, Evans B, Freeman MC, Gordon B, Kang G, Lanata CF, Medlicott KO, Prüss-Ustün A, Troeger C, Boisson S, Cumming O. 2023. Burden of disease attributable to unsafe drinking water, sanitation, and hygiene in domestic settings: a global analysis for selected adverse health outcomes. Lancet 401:2060–2071. doi:10.1016/S0140-6736(23)00458-0 37290458 PMC10290941

[2] World Health Organization. 2023. Typhoid, fact sheets. Available from: https://www.who.int/news-room/fact-sheets/detail/typhoid. Retrieved 10 Dec 2024.

[3] Plumb I, Fields P, Bruce B. 2024. Salmonellosis, nontyphoidal. In CDC yellow book

[4] United States Department of Agriculture - Economic Research Service. 2021. Cost estimates of foodborne illnesses. Available from: https://www.ers.usda.gov/data-products/cost-estimates-of-foodborne-illnesses/. Retrieved 3 Apr 2024.

[5] World Health Organization. 2018. Salmonella (non-typhoidal). Available from: https://www.who.int/news-room/fact-sheets/detail/Salmonella-(non-typhoidal). Retrieved 13 Jan 2025.

[6] IFSAC. 2024. Foodborne illness source attribution estimates – United States, 2022. Available from: https://www.cdc.gov/ifsac/php/data-research/annual-report-2022.html

[7] Bell RL, Kase JA, Harrison LM, Balan KV, Babu U, Chen Y, Macarisin D, Kwon HJ, Zheng J, Stevens EL, Meng J, Brown EW. 2021. The persistence of bacterial pathogens in surface water and its impact on global food safety. Pathogens 10:1391. doi:10.3390/pathogens10111391 34832547 PMC8617848

[8] Centers for Disease Control and Prevention. 2024. Investigation update: Salmonella outbreak, cucumbers - June 2024. Available from: https://www.cdc.gov/salmonella/outbreaks/africana-06-24/investigation.html. Retrieved 19 Feb 2025.

[9] Barton Behravesh C, Mody RK, Jungk J, Gaul L, Redd JT, Chen S, Cosgrove S, Hedican E, Sweat D, Chávez-Hauser L, Snow SL, Hanson H, Nguyen T-A, Sodha SV, Boore AL, Russo E, Mikoleit M, Theobald L, Gerner-Smidt P, Hoekstra RM, Angulo FJ, Swerdlow DL, Tauxe RV, Griffin PM, Williams IT. 2011. 2008 Outbreak of Salmonella Saintpaul infections associated with raw produce. N Engl J Med 364:918–927. doi:10.1056/NEJMoa1005741 21345092

[10] FDA. 2025. Outbreak investigation of Salmonella: cucumbers (May 2025). Available from: https://www.fda.gov/food/outbreaks-foodborne-illness/outbreak-investigation-salmonella-cucumbers-may-2025.html. Retrieved 8 Oct 2025.

[11] Grimont P, Weill F-X. 2007. Antigenic formulae of the Salmonella servovars: WHO collaborating centre for reference and research on Salmonella. 9th ed, p 1–166. Institute Pasteur.

[12] Issenhuth-Jeanjean S, Roggentin P, Mikoleit M, Guibourdenche M, de Pinna E, Nair S, Fields PI, Weill F-X. 2014. Supplement 2008-2010 (no. 48) to the White-Kauffmann-Le Minor scheme. Res Microbiol 165:526–530. doi:10.1016/j.resmic.2014.07.004 25049166

[13] Cheng RA, Eade CR, Wiedmann M. 2019. Embracing diversity: differences in virulence mechanisms, disease severity, and host adaptations contribute to the success of nontyphoidal Salmonella as a foodborne pathogen. Front Microbiol 10:1368. doi:10.3389/fmicb.2019.01368 31316476 PMC6611429

[14] Shah DH, Paul NC, Sischo WC, Crespo R, Guard J. 2017. Population dynamics and antimicrobial resistance of the most prevalent poultry-associated Salmonella serotypes. Poult Sci 96:687–702. doi:10.3382/ps/pew342 27665007

[15] Uzzau S, Brown DJ, Wallis T, Rubino S, Leori G, Bernard S, Casadesús J, Platt DJ, Olsen JE. 2000. Host adapted serotypes of Salmonella enterica. Epidemiol Infect 125:229–255. doi:10.1017/s0950268899004379 11117946 PMC2869595

[16] Gorski L, Noriega AA. 2023. Comparison of phenotype nutritional profiles and phosphate metabolism genes in four serovars of Salmonella enterica from water sources. Microorganisms 11:2109. doi:10.3390/microorganisms11082109 37630669 PMC10459026

[17] Uelze L, Bloch A, Borowiak M, Grobbel M, Deneke C, Fischer M, Malorny B, Pietsch M, Simon S, Szabó I, Tausch SH, Fischer J. 1911. What WGS reveals about Salmonella enterica subsp. enterica in wildlife in Germany. Microorganisms 9:1911. doi:10.3390/microorganisms9091911 PMC8471515 34576806

[18] Patel K, Stapleton GS, Trevejo RT, Tellier WT, Higa J, Adams JK, Hernandez SM, Sanchez S, Nemeth NM, Debess EE, Rogers KH, Mete A, Watson KD, Foss L, Low MSF, Gollarza L, Nichols M. 2023. Human salmonellosis outbreak linked to Salmonella Typhimurium epidemic in wild songbirds, United States, 2020–2021. Emerg Infect Dis 29:2298–2306. doi:10.3201/eid2911.230332 37877570 PMC10617330

[19] Hernandez SM, Welch CN, Peters VE, Lipp EK, Curry S, Yabsley MJ, Sanchez S, Presotto A, Gerner-Smidt P, Hise KB, Hammond E, Kistler WM, Madden M, Conway AL, Kwan T, Maurer JJ. 2016. Urbanized white ibises (Eudocimus albus) as carriers of Salmonella enterica of significance to public health and wildlife. PLoS One 11:e0164402. doi:10.1371/journal.pone.0164402 27768705 PMC5074519

[20] Chiu C-H, Wu T-L, Su L-H, Chu C, Chia J-H, Kuo A-J, Chien M-S, Lin T-Y. 2002. The emergence in Taiwan of fluoroquinolone resistance in Salmonella enterica serotype choleraesuis. N Engl J Med 346:413–419. doi:10.1056/NEJMoa012261 11832529

[21] Cohen E, Azriel S, Auster O, Gal A, Zitronblat C, Mikhlin S, Scharte F, Hensel M, Rahav G, Gal-Mor O. 2021. Pathoadaptation of the passerine-associated Salmonella enterica serovar Typhimurium lineage to the avian host. PLoS Pathog 17:e1009451. doi:10.1371/journal.ppat.1009451 33739988 PMC8011750

[22] Cohen E, Azriel S, Auster O, Gal A, Mikhlin S, Crauwels S, Rahav G, Gal-Mor O. 2024. A new Salmonella enterica serovar that was isolated from a wild sparrow presents a distinct genetic, metabolic and virulence profile. Microbes Infect 26:105249. doi:10.1016/j.micinf.2023.105249 37956735

[23] Marietto-Gonçalves GA, Dias GS, Barbosa CHG, Ribeiro GC, Teixeira ME, Tonin AA, Okamoto AS, Andreatti Filho RL. 2024. Salmonella Typhimurium transmission among free-living birds (Passerines) and broiler chicks. Cad Pedagógico 21:e5925. doi:10.54033/cadpedv21n7-200

[24] Alley MR, Connolly JH, Fenwick SG, Mackereth GF, Leyland MJ, Rogers LE, Haycock M, Nicol C, Reed CEM. 2002. An epidemic of salmonellosis caused by Salmonella Typhimurium DT160 in wild birds and humans in New Zealand. N Z Vet J 50:170–176. doi:10.1080/00480169.2002.36306 16032266

[25] Hughes LA, Shopland S, Wigley P, Bradon H, Leatherbarrow AH, Williams NJ, Bennett M, de Pinna E, Lawson B, Cunningham AA, Chantrey J. 2008. Characterisation of Salmonella enterica serotype Typhimurium isolates from wild birds in northern England from 2005 – 2006. BMC Vet Res 4:4. doi:10.1186/1746-6148-4-4 18230128 PMC2257933

[26] Very KJ, Kirchner MK, Shariat N, Cottrell W, Sandt CH, Dudley EG, Kariyawasam S, Jayarao BM. 2016. Prevalence and spatial distribution of Salmonella infections in the Pennsylvania raccoon (Procyon lotor). Zoonoses Public Health 63:223–233. doi:10.1111/zph.12222 26272724

[27] Maurer JJ, Martin G, Hernandez S, Cheng Y, Gerner-Smidt P, Hise KB, Tobin D’Angelo M, Cole D, Sanchez S, Madden M, Valeika S, Presotto A, Lipp EK. 2015. Diversity and persistence of Salmonella enterica strains in rural landscapes in the southeastern United States. PLoS One 10:e0128937. doi:10.1371/journal.pone.0128937 26131552 PMC4489491

[28] Hernandez SM, Maurer JJ, Yabsley MJ, Peters VE, Presotto A, Murray MH, Curry S, Sanchez S, Gerner-Smidt P, Hise K, Huang J, Johnson K, Kwan T, Lipp EK. 2021. Free-living aquatic turtles as sentinels of Salmonella spp. for water bodies. Front Vet Sci 8:674973. doi:10.3389/fvets.2021.674973 34368271 PMC8339271

[29] Doden G, Gartlan B, Klein K, Maddox CW, Adamovicz LA, Allender MC. 2021. Prevalence and antimicrobial resistance patterns of Salmonella spp. in two free-ranging populations of eastern box turtles (Terrapene carolina carolina). J Zoo Wildl Med 52:863–871. doi:10.1638/2020-0061 34687501

[30] Renter DG, Gnad DP, Sargeant JM, Hygnstrom SE. 2006. Prevalence and serovars of Salmonella in the feces of free-ranging white-tailed deer (Odocoileus virginianus) in Nebraska. J Wildl Dis 42:699–703. doi:10.7589/0090-3558-42.3.699 17092906

[31] Salas-Rosas LM, López González CA, Sánchez-Cervantes A, Vázquez-Peláez CG, Rodríguez-Torres A, Cervantes-Chávez JA, Olvera-Ramírez AM. 2020. Prevalence of Salmonella spp. and Escherichia coli O157 in a red deer herd (Cervus elaphus scoticus) in central Mexico. Appl Anim Sci 36:622–629. doi:10.15232/aas.2020-02032

[32] Topalcengiz Z, Spanninger PM, Jeamsripong S, Persad AK, Buchanan RL, Saha J, LeJEUNE J, Jay-Russell MT, Kniel KE, Danyluk MD. 2020. Survival of Salmonella in various wild animal feces that may contaminate produce. J Food Prot 83:651–660. doi:10.4315/0362-028X.JFP-19-302 32221570

[33] Gorski L, Parker CT, Liang A, Cooley MB, Jay-Russell MT, Gordus AG, Atwill ER, Mandrell RE. 2011. Prevalence, distribution, and diversity of Salmonella enterica in a major produce region of California. Appl Environ Microbiol 77:2734–2748. doi:10.1128/AEM.02321-10 21378057 PMC3126348

[34] Millán J, Aduriz G, Moreno B, Juste RA, Barral M. 2004. Salmonella isolates from wild birds and mammals in the Basque Country (Spain). Rev Sci Tech 23:905–911. doi:10.20506/rst.23.3.1529 15861885

[35] Bell RL, Zheng J, Burrows E, Allard S, Wang CY, Keys CE, Melka DC, Strain E, Luo Y, Allard MW, Rideout S, Brown EW. 2015. Ecological prevalence, genetic diversity, and epidemiological aspects of Salmonella isolated from tomato agricultural regions of the Virginia Eastern Shore. Front Microbiol 6:415. doi:10.3389/fmicb.2015.00415 25999938 PMC4423467

[36] Byappanahalli MN, Sawdey R, Ishii S, Shively DA, Ferguson JA, Whitman RL, Sadowsky MJ. 2009. Seasonal stability of Cladophora-associated Salmonella in Lake Michigan watersheds. Water Res 43:806–814. doi:10.1016/j.watres.2008.11.012 19059626

[37] Santo Domingo JW, Harmon S, Bennett J. 2000. Survival of Salmonella species in river water. Curr Microbiol 40:409–417. doi:10.1007/s002840010079 10827285

[38] Gaertner JP, Mendoza JA, Forstner MRJ, Hahn D. 2011. Recovery of Salmonella from biofilms in a headwater spring ecosystem. J Water Health 9:458–466. doi:10.2166/wh.2011.173 21976193

[39] Li B, Jackson SA, Gangiredla J, Wang W, Liu H, Tall BD, Beaubrun JJ-G, Jay-Russell M, Vellidis G, Elkins CA. 2015. Genomic evidence reveals numerous Salmonella enterica serovar Newport reintroduction events in Suwannee watershed irrigation ponds. Appl Environ Microbiol 81:8243–8253. doi:10.1128/AEM.02179-15 26386063 PMC4644655

[40] Liu H, Whitehouse CA, Li B. 2018. Presence and persistence of Salmonella in water: the impact on microbial quality of water and food safety. Front Public Health 6:159. doi:10.3389/fpubh.2018.00159 29900166 PMC5989457

[41] Sha Q, Vattem DA, Forstner MRJ, Hahn D. 2013. Quantifying Salmonella population dynamics in water and biofilms. Microb Ecol 65:60–67. doi:10.1007/s00248-012-0106-y 22890729

[42] Topalcengiz Z, McEgan R, Danyluk MD. 2019. Fate of Salmonella in central Florida surface waters and evaluation of EPA worst case water as a standard medium. J Food Prot 82:916–925. doi:10.4315/0362-028X.JFP-18-331 31081689

[43] Brandl MT, Rosenthal BM, Haxo AF, Berk SG. 2005. Enhanced survival of Salmonella enterica in vesicles released by a soilborne Tetrahymena species. Appl Environ Microbiol 71:1562–1569. doi:10.1128/AEM.71.3.1562-1569.2005 15746361 PMC1065168

[44] Micallef SA, Rosenberg Goldstein RE, George A, Kleinfelter L, Boyer MS, McLaughlin CR, Estrin A, Ewing L, Jean-Gilles Beaubrun J, Hanes DE, Kothary MH, Tall BD, Razeq JH, Joseph SW, Sapkota AR. 2012. Occurrence and antibiotic resistance of multiple Salmonella serotypes recovered from water, sediment and soil on mid-Atlantic tomato farms. Environ Res 114:31–39. doi:10.1016/j.envres.2012.02.005 22406288

[45] Gorski L, Liang AS, Walker S, Carychao D, Aviles Noriega A, Mandrell RE, Cooley MB. 2022. Salmonella enterica serovar diversity, distribution, and prevalence in public-access waters from a central California coastal leafy green-growing region from 2011 to 2016. Appl Environ Microbiol 88:e0183421. doi:10.1128/AEM.01834-21 34910555 PMC8824205

[46] Deaven AM, Ferreira CM, Reed EA, Chen See JR, Lee NA, Almaraz E, Rios PC, Marogi JG, Lamendella R, Zheng J, Bell RL, Shariat NW. 2021. Salmonella genomics and population analyses reveal high inter- and intraserovar diversity in freshwater. Appl Environ Microbiol 87:1–14. doi:10.1128/AEM.02594-20 PMC8104997 33397693

[47] Chen Z, Moreno-Switt AI, Reyes-Jara A, Delgado Suarez E, Adell AD, Oliveira CJB, Bonelli RR, Huang X, Brown E, Allard M, Grim C, Bell R, Meng J, Toro M. 2024. A multicenter genomic epidemiological investigation in Brazil, Chile, and Mexico reveals the diversity and persistence of Salmonella populations in surface waters. mBio 15:e0077724. doi:10.1128/mbio.00777-24 38920393 PMC11253603

[48] Gorski L, Shariat NW, Richards AK, Siceloff AT, Aviles Noriega A, Harhay DM. 2024. Growth assessment of Salmonella enterica multi-serovar populations in poultry rinsates with commonly used enrichment and plating media. Food Microbiol 119:104431. doi:10.1016/j.fm.2023.104431 38225041

[49] Thompson CP, Doak AN, Amirani N, Schroeder EA, Wright J, Kariyawasam S, Lamendella R, Shariat NW. 2018. High-resolution identification of multiple Salmonella serovars in a single sample by using CRISPR-SeroSeq. Appl Environ Microbiol 84:1–13. doi:10.1128/AEM.01859-18 PMC6193385 30170999

[50] Haley BJ, Cole DJ, Lipp EK. 2009. Distribution, diversity, and seasonality of waterborne salmonellae in a rural watershed. Appl Environ Microbiol 75:1248–1255. doi:10.1128/AEM.01648-08 19124594 PMC2648171

[51] McEgan R, Mootian G, Goodridge LD, Schaffner DW, Danyluk MD. 2013. Predicting Salmonella populations from biological, chemical, and physical indicators in Florida surface waters. Appl Environ Microbiol 79:4094–4105. doi:10.1128/AEM.00777-13 23624476 PMC3697547

[52] Murphy CM, Strawn LK, Chapin TK, McEgan R, Gopidi S, Friedrich L, Goodridge LD, Weller DL, Schneider KR, Danyluk MD. 2022. Factors associated with E. coli levels in and Salmonella contamination of agricultural water differed between north and south Florida waterways. Front Water 3. doi:10.3389/frwa.2021.750673

[53] Weller D, Belias A, Green H, Roof S, Wiedmann M. 2020. Landscape, water quality, and weather factors associated with an increased likelihood of foodborne pathogen contamination of New York streams used to source water for produce production. Front Sustain Food Syst 3:124. doi:10.3389/fsufs.2019.00124 32440656 PMC7241490

[54] Truitt LN, Vazquez KM, Pfuntner RC, Rideout SL, Havelaar AH, Strawn LK. 2018. Microbial quality of agricultural water used in produce preharvest production on the eastern shore of Virginia. J Food Prot 81:1661–1672. doi:10.4315/0362-028X.JFP-18-185 30212229

[55] Centers for Disease Control and Prevention. BEAM (Bacteria, Enterics, Amoeba, and Mycotics) dashboard. Available from: www.cdc.gov/ncezid/dfwed/BEAM-dashboard.html. Retrieved 03 Apr 2024.

[56] Cox NA, Berrang ME, House SL, Medina D, Cook KL, Shariat NW. 2019. Population analyses reveal preenrichment method and selective enrichment media affect Salmonella serovars detected on broiler carcasses. J Food Prot 82:1688–1696. doi:10.4315/0362-028X.JFP-19-166 31536420

[57] Dieter CA, Maupin MA, Caldwell RR, Harris MA, Ivahnenko TI, Lovelace JK, Barber NL, Linsey KS. 2018. Estimated use of water in the United States in 2015

[58] Laborda P, Sanz-García F, Ochoa-Sánchez LE, Gil-Gil T, Hernando-Amado S, Martínez JL. 2022. Wildlife and antibiotic resistance. Front Cell Infect Microbiol 12:873989. doi:10.3389/fcimb.2022.873989 35646736 PMC9130706

[59] Ramey AM, Ahlstrom CA. 2020. Antibiotic resistant bacteria in wildlife: perspectives on trends, acquisition and dissemination, data gaps, and future directions. J Wildl Dis 56:1–15. doi:10.7589/2019-04-099 31567035

[60] Carroll D, Wang J, Fanning S, McMahon BJ. 2015. Antimicrobial resistance in wildlife: implications for public health. Zoonoses Public Health 62:534–542. doi:10.1111/zph.12182 25639901

[61] Espunyes J, Cabezón O, Dias-Alves A, Miralles P, Ayats T, Cerdà-Cuéllar M. 2021. Assessing the role of livestock and sympatric wild ruminants in spreading antimicrobial resistant Campylobacter and Salmonella in alpine ecosystems. BMC Vet Res 17:79. doi:10.1186/s12917-021-02784-2 33588859 PMC7885356

[62] McMillan EA, Jackson CR, Frye JG. 2020. Transferable plasmids of Salmonella enterica associated with antibiotic resistance genes. Front Microbiol 11. doi:10.3389/fmicb.2020.562181 PMC7578388 33133037

[63] Elsohaby I, Samy A, Elmoslemany A, Alorabi M, Alkafafy M, Aldoweriej A, Al-Marri T, Elbehiry A, Fayez M. 2021. Migratory wild birds as a potential disseminator of antimicrobial-resistant bacteria around Al-Asfar Lake, Eastern Saudi Arabia. Antibiotics (Basel) 10:260. doi:10.3390/antibiotics10030260 33807576 PMC8000645

[64] Zhao H, Sun R, Yu P, Alvarez PJJ. 2020. High levels of antibiotic resistance genes and opportunistic pathogenic bacteria indicators in urban wild bird feces. Environ Pollut 266:115200. doi:10.1016/j.envpol.2020.115200 32663725

[65] Karp DS, Gennet S, Kilonzo C, Partyka M, Chaumont N, Atwill ER, Kremen C. 2015. Comanaging fresh produce for nature conservation and food safety. Proc Natl Acad Sci USA 112:11126–11131. doi:10.1073/pnas.1508435112 26261343 PMC4568220

[66] Baur P, Getz C, Sowerwine J. 2017. Contradictions, consequences and the human toll of food safety culture. Agric Hum Values 34:713–728. doi:10.1007/s10460-017-9772-1

[67] Adalja A, Lichtenberg E. 2018. Produce growers’ cost of complying with the Food Safety Modernization Act. Food Policy 74:23–38. doi:10.1016/j.foodpol.2017.10.005

[68] Devarajan N, Weller DL, Jones M, Adell AD, Adhikari A, Allende A, Arnold NL, Baur P, Beno SM, Clements D, et al.. 2023. Evidence for the efficacy of pre-harvest agricultural practices in mitigating food-safety risks to fresh produce in North America. Front Sustain Food Syst 7:1101435. doi:10.3389/fsufs.2023.1101435

[69] Weller D, Brassill N, Rock C, Ivanek R, Mudrak E, Roof S, Ganda E, Wiedmann M. 2020. Complex interactions between weather, and microbial and physicochemical water quality impact the likelihood of detecting foodborne pathogens in agricultural water. Front Microbiol 11:134. doi:10.3389/fmicb.2020.00134 32117154 PMC7015975

[70] Chung T, Yan R, Weller DL, Kovac J. 2023. Conditional forest models built using metagenomic data accurately predicted Salmonella contamination in northeastern streams. Microbiol Spectr 11:e0038123. doi:10.1128/spectrum.00381-23 36946722 PMC10100987

[71] Cho S., Jackson CR, Frye JG. 2020. The prevalence and antimicrobial resistance phenotypes of Salmonella, Escherichia coli and Enterococcus sp. in surface water. Lett Appl Microbiol 71:3–25. doi:10.1111/lam.13301 32304575

[72] Cho S, Hiott LM, House SL, Woodley TA, McMillan EA, Sharma P, Barrett JB, Adams ES, Brandenburg JM, Hise KB, Bateman McDonald JM, Ottesen EA, Lipp EK, Jackson CR, Frye JG. 2022. Analysis of Salmonella enterica isolated from a mixed-use watershed in Georgia, USA: antimicrobial resistance, serotype diversity, and genetic relatedness to human isolates. Appl Environ Microbiol 88:e0039322. doi:10.1128/aem.00393-22 35532233 PMC9128517

[73] Strawn LK, Fortes ED, Bihn EA, Nightingale KK, Gröhn YT, Worobo RW, Wiedmann M, Bergholz PW. 2013. Landscape and meteorological factors affecting prevalence of three food-borne pathogens in fruit and vegetable farms. Appl Environ Microbiol 79:588–600. doi:10.1128/AEM.02491-12 23144137 PMC3553790

[74] Toro M, Weller D, Ramos R, Diaz L, Alvarez FP, Reyes-Jara A, Moreno-Switt AI, Meng J, Adell AD. 2022. Environmental and anthropogenic factors associated with the likelihood of detecting Salmonella in agricultural watersheds. Environ Pollut 306:119298. doi:10.1016/j.envpol.2022.119298 35430308

[75] Vereen E Jr, Lowrance RR, Jenkins MB, Adams P, Rajeev S, Lipp EK. 2013. Landscape and seasonal factors influence Salmonella and Campylobacter prevalence in a rural mixed use watershed. Water Res 47:6075–6085. doi:10.1016/j.watres.2013.07.028 23969398

[76] Sharma M, Handy ET, East CL, Kim S, Jiang C, Callahan MT, Allard SM, Micallef S, Craighead S, Anderson-Coughlin B, et al.. 2020. Prevalence of Salmonella and Listeria monocytogenes in non-traditional irrigation waters in the Mid-Atlantic United States is affected by water type, season, and recovery method. PLoS One 15:e0229365. doi:10.1371/journal.pone.0229365 32182252 PMC7077874

[77] Gu G, Strawn LK, Ottesen AR, Ramachandran P, Reed EA, Zheng J, Boyer RR, Rideout SL. 2020. Correlation of Salmonella enterica and Listeria monocytogenes in irrigation water to environmental factors, fecal indicators, and bacterial communities. Front Microbiol 11:557289. doi:10.3389/fmicb.2020.557289 33488530 PMC7820387

[78] Murphy CM, Weller DL, Strawn LK. 2024. Scale and detection method impacted Salmonella prevalence and diversity in ponds. Sci Total Environ 907:167812. doi:10.1016/j.scitotenv.2023.167812 37852489

[79] Lima LA, Rocha ADL, Gomes MLR, Pereira WE, Givisiez PEN, Brown EW, Allard MW, Chen Z, Bell RL, Toro M, Meng J, Oliveira CJB. 2025. Water sampling using modified Moore swab (MMS): the effects of sampling replicates and different media on the frequency and diversity of Salmonella serovars. Appl Environ Microbiol 91:e0064725. doi:10.1128/aem.00647-25 40704814 PMC12366358

[80] da Silva KE, Yokoyama T, Naga SR, Maharjan M, Pereira Dos Santos PC, Fisher KN, Coulibaly JT, Yang MZ, Nelson EJ, Charles RC, Shafer K, Igiraneza B-L, Yusuf S, Mulder E, Neuzil K, Bogoch II, Shrestha R, Tamrakar D, Andrews JR. 2025. Rapid, low-cost colorimetric detection of Salmonella Typhi bacteriophages for environmental surveillance. mBio 16:e0196325. doi:10.1128/mbio.01963-25 40823833 PMC12421811

[81] Kim S, Pachepsky Y, Micallef SA, Rosenberg Goldstein R, Sapkota AR, Hashem F, Parveen S, Kniel KE, Sharma M. 2023. Temporal stability of Salmonella enterica and Listeria monocytogenes in surface waters used for irrigation in the mid-Atlantic United States. J Food Prot 86:100058. doi:10.1016/j.jfp.2023.100058 37005038

[82] Gruszynski K, Pao S, Kim C, Toney D, Wright K, Ross PG, Colon A, Levine S. 2014. Evaluating wildlife as a potential source of Salmonella serotype Newport (JJPX01.0061) contamination for tomatoes on the eastern shore of Virginia. Zoonoses Public Health 61:202–207. doi:10.1111/zph.12061 23773825

[83] Fakruddin M, Mannan KSB, Andrews S. 2013. Viable but nonculturable bacteria: food safety and public health perspective. ISRN Microbiol 2013:703813. doi:10.1155/2013/703813 24191231 PMC3804398

[84] Deaven AM, Ferreira CM, Reed EA, Chen See JR, Lee NA, Almaraz E, Rios PC, Marogi JG, Lamendella R, Zheng J, Bell RL, Shariat NW. 2021. Salmonella genomics and population analyses reveal high inter- and intraserovar diversity in freshwater. Appl Environ Microbiol 87:e02594-20. doi:10.1128/AEM.02594-20 33397693 PMC8104997

[85] Teachey ME, McDonald JM, Ottesen EA. 2019. Rapid and stable microbial community assembly in the headwaters of a third-order stream. Appl Environ Microbiol 85:e00188-19. doi:10.1128/AEM.00188-19 30952660 PMC6532045

[86] Liu B, Wang Y, Zhang H, Zhou Y, Zhang C, Yang N, Wang W. 2023. The variations of microbial diversity and community structure along different stream orders in Wuyi Mountains. Microb Ecol 86:2330–2343. doi:10.1007/s00248-023-02240-8 37222804

[87] Cason J, Cox N, Buhr R, Bourassa D, Richardson L. 2011. Probability of identifying different *Salmonella* serotypes in poultry samples, p 75–76. In International poultry science forum

[88] Siceloff AT, Waltman D, Shariat NW. 2022. Regional Salmonella differences in United States broiler production from 2016 to 2020 and the contribution of multiserovar populations to Salmonella surveillance. Appl Environ Microbiol 88:e0020422. doi:10.1128/aem.00204-22 35384708 PMC9040615

[89] Obe T, Siceloff AT, Crowe MG, Scott HM, Shariat NW. 2023. Combined quantification and deep serotyping for Salmonella risk profiling in broiler flocks. Appl Environ Microbiol 89:e0203522. doi:10.1128/aem.02035-22 36920215 PMC10132105

[90] Siceloff AT, Ohta N, Norman KN, Loneragan GH, Norby B, Scott HM, Shariat NW. 2021. Antimicrobial resistance hidden within multiserovar Salmonella populations. Antimicrob Agents Chemother 65:1–6. doi:10.1128/AAC.00048-21 PMC8315986 33782004

[91] Smith JC, Varriano S, Roach K, Snipes Z, Dawson JL, Shealy J, Dunn LL, Snyder WE, Shariat NW. 2023. Prevalence and molecular characterization of Salmonella isolated from wild birds in fresh produce environments. Front Microbiol 14:1272916. doi:10.3389/fmicb.2023.1272916 38029194 PMC10662084

[92] Cason EE, Carlson AV, Siemens AL, Shariat NW. 2024. High-resolution serotyping reveals Salmonella surveillance challenges in the Turkey industry. J Food Prot 87:100319. doi:10.1016/j.jfp.2024.100319 38908798

[93] Lipman DJ, Cherry JL, Strain E, Agarwala R, Musser SM. 2024. Genomic perspectives on foodborne illness. Proc Natl Acad Sci USA 121:e2411894121. doi:10.1073/pnas.2411894121 39499629 PMC11573619

[94] Berge ACB, Dueger EL, Sischo WM. 2006. Comparison of Salmonella enterica serovar distribution and antibiotic resistance patterns in wastewater at municipal water treatment plants in two California cities. J Appl Microbiol 101:1309–1316. doi:10.1111/j.1365-2672.2006.03031.x 17105561

[95] M’ikanatha NM, Goldblum ZS, Cesari N, Nawrocki EM, Fu Y, Kovac J, Dudley EG. 2024. Outbreak-associated Salmonella Baildon found in wastewater demonstrates how sewage monitoring can supplement traditional disease surveillance. J Clin Microbiol 62:e0082524. doi:10.1128/jcm.00825-24 39297648 PMC11481576

[96] Diemert S, Yan T. 2019. Clinically unreported salmonellosis outbreak detected via comparative genomic analysis of municipal wastewater Salmonella isolates. Appl Environ Microbiol 85:e00139-19. doi:10.1128/AEM.00139-19 30902850 PMC6498150

[97] Diemert S, Yan T. 2020. Municipal wastewater surveillance revealed a high community disease burden of a rarely reported and possibly subclinical Salmonella enterica serovar Derby strain. Appl Environ Microbiol 86:1–12. doi:10.1128/AEM.00814-20 PMC7440783 32591375

[98] Vincent V, Scott HM, Harvey RB, Alali WQ, Hume ME. 2007. Novel surveillance of Salmonella enterica serotype Heidelberg epidemics in a closed community. Foodborne Pathog Dis 4:375–385. doi:10.1089/fpd.2007.0025 17883321

[99] Waltenburg MA, Perez A, Salah Z, Karp BE, Whichard J, Tolar B, Gollarza L, Koski L, Blackstock A, Basler C, Nichols M. 2022. Multistate reptile- and amphibian-associated salmonellosis outbreaks in humans, United States, 2009-2018. Zoonoses Public Health 69:925–937. doi:10.1111/zph.12990 36345968 PMC9804608

[100] Pees M, Brockmann M, Steiner N, Marschang RE. 2023. Salmonella in reptiles: a review of occurrence, interactions, shedding and risk factors for human infections. Front Cell Dev Biol 11:1251036. doi:10.3389/fcell.2023.1251036 37822870 PMC10562597

[101] Rocha AD de L, Ferrari RG, Pereira WE, Lima LA de, Givisiez PEN, Moreno-Switt AI, Toro M, Delgado-Suárez EJ, Meng J, Oliveira CJB de. 2022. Revisiting the biological behavior of Salmonella enterica in hydric resources: a meta-analysis study addressing the critical role of environmental water on food safety and public health. Front Microbiol 13. doi:10.3389/fmicb.2022.802625 PMC9201643 35722289

[102] Rajabi M, Jones M, Hubbard M, Rodrick G, Wright AC. 2011. Distribution and genetic diversity of Salmonella enterica in the upper Suwannee River. Int J Microbiol 2011:461321. doi:10.1155/2011/461321 22347228 PMC3278925

[103] Al Mawly J, Grinberg A, Prattley D, Moffat J, French N. 2015. Prevalence of endemic enteropathogens of calves in New Zealand dairy farms. N Z Vet J 63:147–152. doi:10.1080/00480169.2014.966168 25237728

[104] Siceloff AT, Ohta N, Norman KN, Loneragan GH, Norby B, Scott HM, Shariat NW. 2021. Antimicrobial resistance hidden within multiserovar Salmonella populations. Antimicrob Agents Chemother 65:e00048-21. doi:10.1128/AAC.00048-21 33782004 PMC8315986

[105] McMillan EA, Weinroth MD, Frye JG. 2022. Increased prevalence of Salmonella Infantis isolated from raw chicken and Turkey products in the United States is due to a single clonal lineage carrying the pESI plasmid. Microorganisms 10:1478. doi:10.3390/microorganisms10071478 35889197 PMC9318337

[106] McMillan EA, Wasilenko JL, Tagg KA, Chen JC, Simmons M, Gupta SK, Tillman GE, Folster J, Jackson CR, Frye JG. 2020. Carriage and gene content variability of the pesi-like plasmid associated with Salmonella Infantis recently established in United States poultry production. Genes (Basel) 11:1–15. doi:10.3390/genes11121516 PMC7766811 33352984

[107] Moore B. 1948. The detection of paratyphoid carriers in towns by means of sewage examination. Mon Bull Min Hlth Lond 7:241–248.

[108] Love MI, Huber W, Anders S. 2014. Moderated estimation of fold change and dispersion for RNA-seq data with DESeq2. Genome Biol 15:550. doi:10.1186/s13059-014-0550-8 25516281 PMC4302049

[109] King RS, Baker ME, Whigham DF, Weller DE, Jordan TE, Kazyak PF, Hurd MK. 2005. Spatial considerations for linking watershed land cover to ecological indicators in streams. Ecol Appl 15:137–153. doi:10.1890/04-0481

[110] CLSI. 2024. Performance standards for antimicrobial susceptibility testing. 34th ed

[111] The National Center for Biotechnology Information. Pathogen detection. Retrieved 08 Jun 2023.

[112] Page AJ, Cummins CA, Hunt M, Wong VK, Reuter S, Holden MTG, Fookes M, Falush D, Keane JA, Parkhill J. 2015. Roary: rapid large-scale prokaryote pan genome analysis. Bioinformatics 31:3691–3693. doi:10.1093/bioinformatics/btv421 26198102 PMC4817141

[113] Piñeiro C, Abuín JM, Pichel JC. 2020. Very Fast Tree: speeding up the estimation of phylogenies for large alignments through parallelization and vectorization strategies. Bioinformatics 36:4658–4659. doi:10.1093/bioinformatics/btaa582 32573652

[114] Letunic I, Bork P. 2021. Interactive Tree Of Life (iTOL) v5: an online tool for phylogenetic tree display and annotation. Nucleic Acids Res 49:W293–W296. doi:10.1093/nar/gkab301 33885785 PMC8265157

[115] Kozlov AM, Darriba D, Flouri T, Morel B, Stamatakis A. 2019. RAxML-NG: a fast, scalable and user-friendly tool for maximum likelihood phylogenetic inference. Bioinformatics 35:4453–4455. doi:10.1093/bioinformatics/btz305 31070718 PMC6821337

[116] Bankevich A, Nurk S, Antipov D, Gurevich AA, Dvorkin M, Kulikov AS, Lesin VM, Nikolenko SI, Pham S, Prjibelski AD, Pyshkin AV, Sirotkin AV, Vyahhi N, Tesler G, Alekseyev MA, Pevzner PA. 2012. SPAdes: a new genome assembly algorithm and its applications to single-cell sequencing. J Comput Biol 19:455–477. doi:10.1089/cmb.2012.0021 22506599 PMC3342519

[117] Zhou Z., Charlesworth J, Achtman M. 2021. HierCC: a multi-level clustering scheme for population assignments based on core genome MLST. Bioinformatics 37:3645–3646. doi:10.1093/bioinformatics/btab234 33823553 PMC8545296

[118] Zhou Z, Alikhan N-F, Sergeant MJ, Luhmann N, Vaz C, Francisco AP, Carriço JA, Achtman M. 2018. GrapeTree: visualization of core genomic relationships among 100,000 bacterial pathogens. Genome Res 28:1395–1404. doi:10.1101/gr.232397.117 30049790 PMC6120633

[119] Zhou Z, Alikhan NF, Mohamed K, Fan Y, Achtman M. 2020. The EnteroBase user’s guide, with case studies on Salmonella transmissions, Yersinia pestis phylogeny, and Escherichia core genomic diversity. Genome Res 30:138–152. doi:10.1101/gr.251678.119 31809257 PMC6961584

[120] Davis S, Pettengill JB, Luo Y, Payne J, Shpuntoff A, Rand H, Strain E. 2015. CFSAN SNP Pipeline: an automated method for constructing SNP matrices from next-generation sequence data. PeerJ Comput Sci 1:e20. doi:10.7717/peerj-cs.20

