# Supplementary material for: A two-year study of Salmonella in four natural watersheds highlights the need for increased environmental Salmonella surveillance to close the One Health loop
Source: Appl Environ Microbiol. 2025 Dec 4;91(12):e01770-25. doi: 10.1128/aem.01770-25 (PMC12724317; doi:10.1128/aem.01770-25)

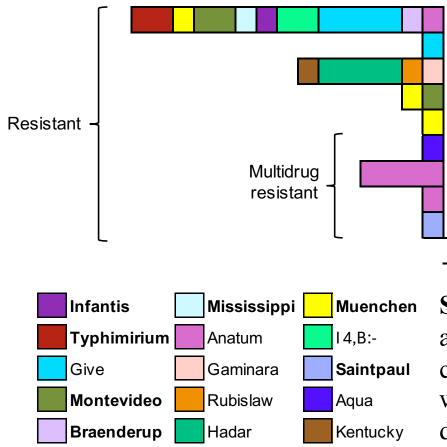

Watershed A	Watershed B	Watershed C	Watershed D	Antibiotic Resistance Profile
80	39	102	60	Pansusceptible (281)
7	2	4	2	Streptomycin (15)
1				Tetracycline (1)
3	2		2	Streptomycin,Tetracycline (7)
		1	1	Tetracycline, Chloramphenicol (2)
			1	Streptomycin,Tetracyclin, Nalidixic Acid (1)
			1	Tetracycline, Chloramphenicol, Amoxicillin/Clavulanic Acid (1)
		4		Ampicillin, Streptomycin, Tetracycline, Chloramphenicol (4)
		1		Ampicillin, Amoxicillin/Clavulanic Acid, Streptomycin, Tetracycline, Chloramphenicol (1)
	1			Ampicillin, Ceftriaxone, Gentamicin, Streptomycin, Tetracycline, Sulfamethoxazole-Trimethoprim, Chloramphenicol (1)
91	44	112	67	

Supplemental Figure 1. Assessment of antibiotic-resistant *Salmonella* isolates. Kirby-Bauer disk diffusion assays were used to assess resistance to ampicillin, amoxicillin-clavulanic acid, ceftriaxone, gentamicin, streptomycin, tetracycline, ciprofloxacin, sulfamethoxazole-trimethoprim, and chloramphenicol. The classification of resistance was determined by CLSI, and an intermediate result was considered as susceptible. Multidrug resistance was defined by resistance to three of more classes of antibiotics. Serovar identification was completed using SeqSero from whole genome sequencing data. Bolded serovars are of highest human clinical importance as denoted by their presence in the top 15 serovars listed on the BEAM Dashboard.

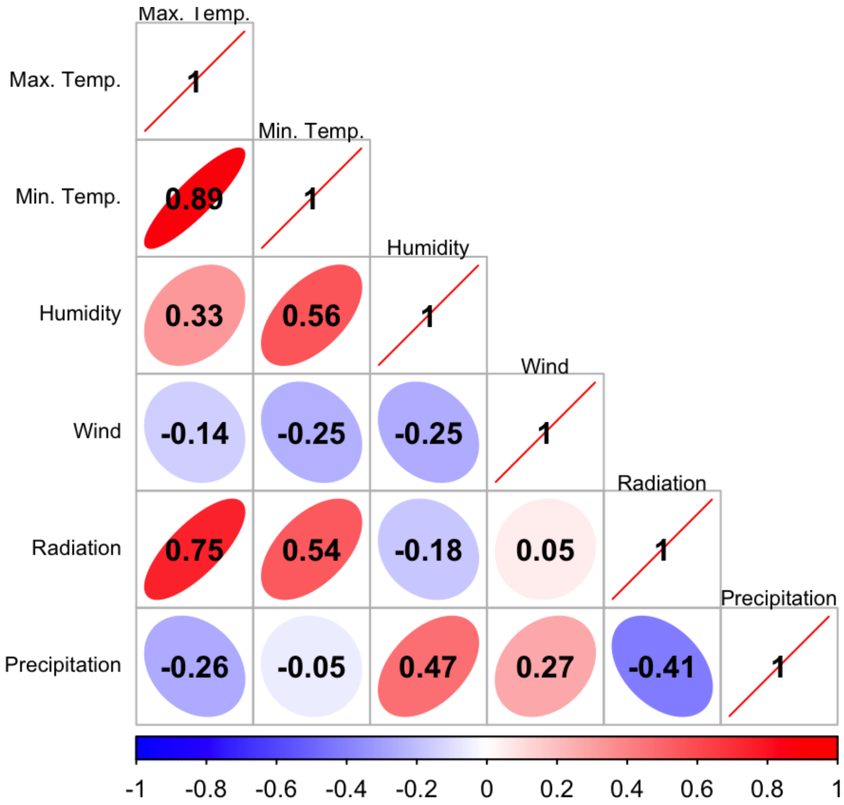

Supplemental Figure 2. Association between different weather variables. Pearson correlation coefficient was calculated for each pairwise combination of weather variables recorded in this study. The resulting coefficients are displayed within each respective box of the matrix, with the size of the circle indicating the strength and direction of association and the color denoting if it was positive (red) or negative (blue).

Supplemental Figure 3. *Salmonella*-positive water samples contain high serovar diversity. CRISPR-SeroSeq was used to determine the relative abundance of *Salmonella* serovars within each sample, where results from all positive enrichments (BPW, RV, TT) were normalized and combined. Samples are arranged according to the system, season, and site they were collected in, with fall in the first year of the study split between 2021 and 2022. The individual serovars are shown on the top, number of serovars identified per sample included on the right, and the heatmap shows the relative serovar abundance in each sample according to the key. Labels containing two serovars reflects that it is not possible to differentiate based on deep serotyping alone.

See supplementary Excel file for full figure

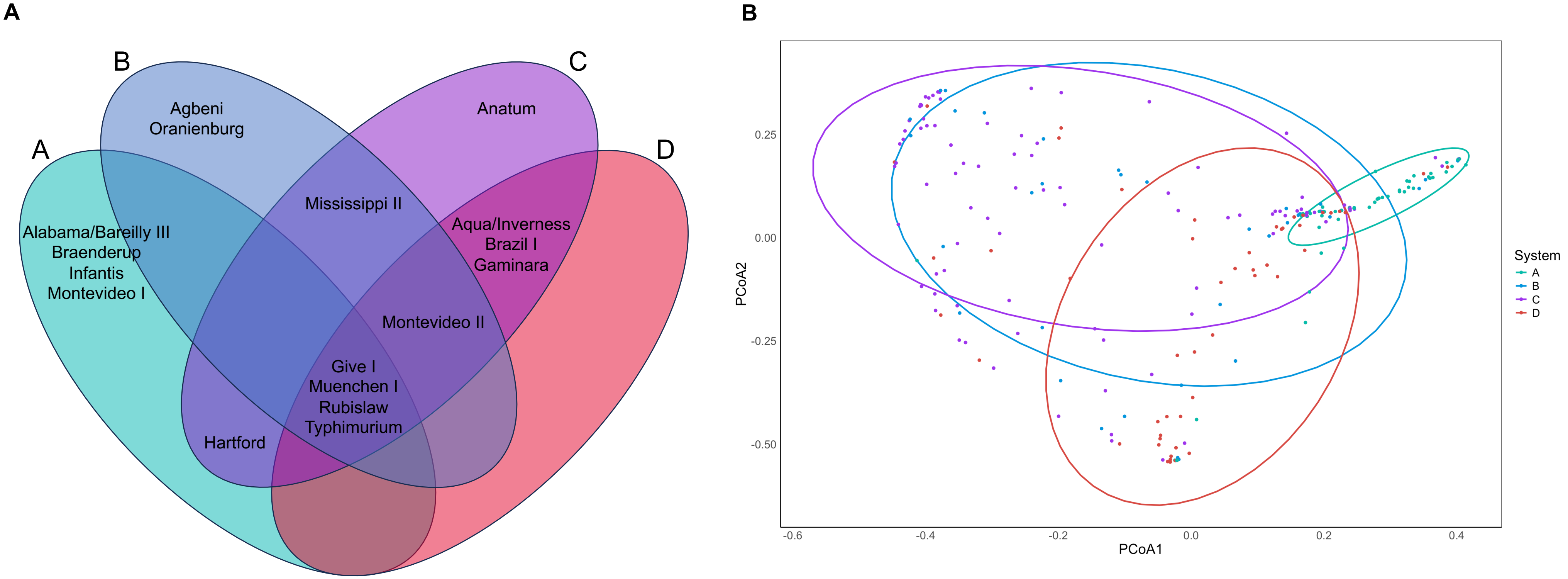

Supplemental Figure 4. Distribution of serovars and serovar populations across the watersheds. A) Venn diagram displays the overlap of serovars found at least five times in each respective watershed. Serovars located only within one ellipse were only found five or more times in that watershed but could have been identified four or less times in any other watershed. B) Principal component analysis of the Jaccard distance of samples within watersheds is shown with ellipses containing 75% of each watershed's samples. A larger ellipse indicates a more variable population, while a smaller ellipse indicates higher consistency.

Supplemental Figure 5. Preliminary serovar Infantis phylogenies including nine isolates from this study and publicly available genomes from NCBI. Study isolates are indicated with a green bar, with the label format of month-watershed-site, and NCBI isolates are listed by sample ID. The phylogenies are rooted at the midpoint. A) Phylogeny includes one representative isolate from each available SNP cluster belonging to serovar Infantis on NCBI (n = 650). Yellow highlight indicates clade selection for subsequent phylogeny. B) Phylogeny includes one representative isolate from the most closely related SNP clusters (n = 30). Blue highlight indicates clade selection for subsequent phylogeny.

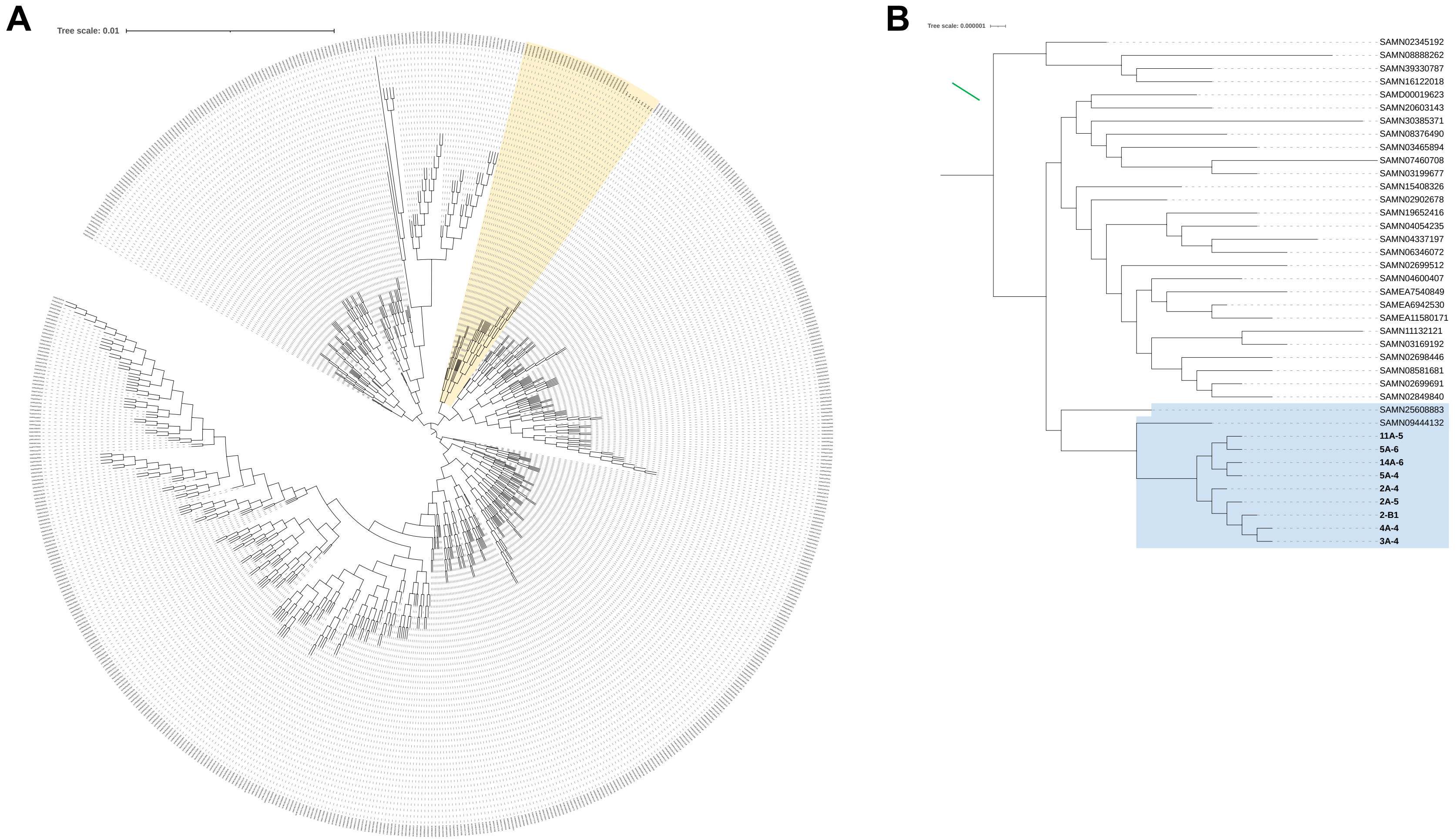

Supplemental Figure 6. Preliminary serovar Typhimurium phylogenies including seven isolates from this study and publicly available genomes from NCBI. Study isolates are bolded, with the label format of month-watershed-site, and NCBI isolates are listed by sample ID. The phylogenies are rooted at the midpoint. A) Phylogeny includes one representative isolate from each available SNP cluster belonging to serovar Typhimurium on NCBI (n = 3,057). Yellow highlight indicates clade selection for subsequent phylogeny. B) Phylogeny includes one representative isolate from the most closely related SNP clusters (n = 20). Blue highlight indicates clade selection for subsequent phylogeny.

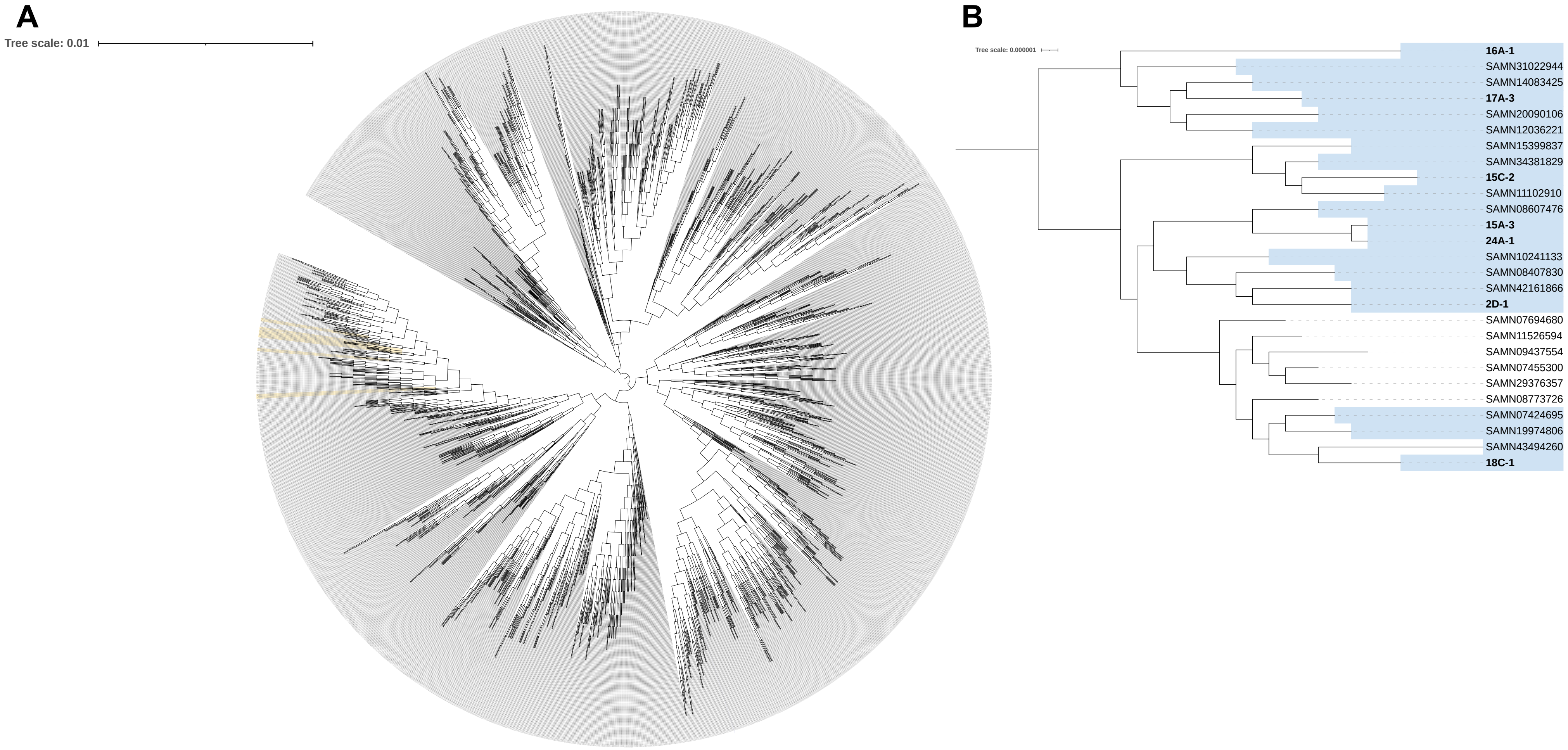

Supplement: Supplemental figures — Figures S1 to S6. [file aem.01770-25-s0001.pdf]
